# Native mass spectrometry reveals DltA catalysis, DltC loading, and inhibition in the d-alanylation pathway

**DOI:** 10.1039/d6ra04213a

**Published:** 2026-06-02

**Authors:** Yi Wang, Hannah Squires, Marian Aba Addo, Jospeh P. Gerdt, Tarick J. El-Baba, R. Craig MacLean, Carol V. Robinson, Jani R. Bolla

**Affiliations:** a Department of Biochemistry, University of Oxford Oxford OX1 3QU UK jani.bolla@bioch.ox.ac.uk; b Department of Chemistry, Indiana University Bloomington Indiana 47405-7012 USA; c Department of Chemistry, University of Oxford Oxford OX1 3QZ UK; d Kavli Institute for Nanoscience Discovery, University of Oxford Oxford OX1 3QU UK; e Department of Biology, University of Oxford Oxford OX1 3RB UK

## Abstract

Gram-positive bacteria protect their cell envelope through d-alanylation of lipoteichoic acid (LTA), a process initiated by DltA-mediated activation of d-alanine and loading onto the carrier protein DltC. Although structural and biochemical studies have established key features of DltA catalysis, direct observation of adenylate formation and carrier protein loading within a reconstituted DltA–DltC system has remained limited. Here, we reconstituted the *Bacillus subtilis*d-alanylation pathway and used native mass spectrometry to resolve DltA-dependent reaction intermediates and products. We detected ATP-dependent adenylation of d-alanine by DltA followed by transfer to *holo*-DltC. This process was inhibited by a sulfamoyl-adenosine compound that mimics the adenylate intermediate. Mutational analysis of the conserved DltA P-loop revealed position-specific effects on catalysis, highlighting structural features that govern substrate processing. Together, these findings define regulatory steps in the Dlt pathway and identify opportunities for targeted inhibition. The same strategy should be adaptable to other systems in which transient acyl- or aminoacyl-carrier protein intermediates are difficult to monitor directly.

## Abstract 2

Gram-positive bacteria protect their cell envelope by d-alanylating lipoteichoic acid (LTA). DltA activates d-alanine and loads it onto *holo*-DltC, which works with DltB, DltX and DltD to transfer d-alanine onto LTA.

## Graphical abstract

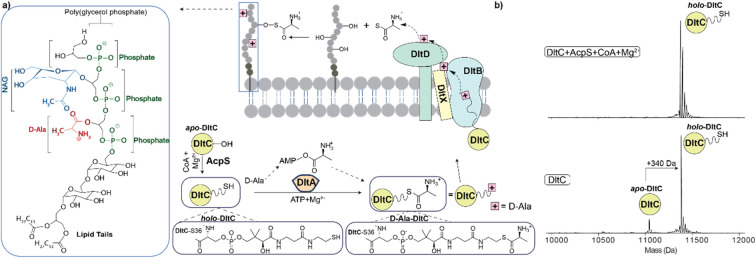

## Introduction

Teichoic acids (TAs) are essential components of almost all Gram-positive bacteria and are anchored either to the cytoplasmic membrane (lipoteichoic acids, LTAs) or to the peptidoglycan layer (wall teichoic acids, WTAs).^1^ TAs contribute to cation homeostasis, host interactions, and biofilm formation.^2–4^ Both LTAs and WTAs are often modified with d-alanine esters, which reduce the net negative charge of the cell envelope and improve bacterial fitness under stress conditions, particularly in the presence of host-derived cationic antimicrobial peptides.^5^d-alanylation also contributes to resistance against lysozyme, and other components of innate immunity, and promotes host colonisation.^6^

**Fig. 1:**
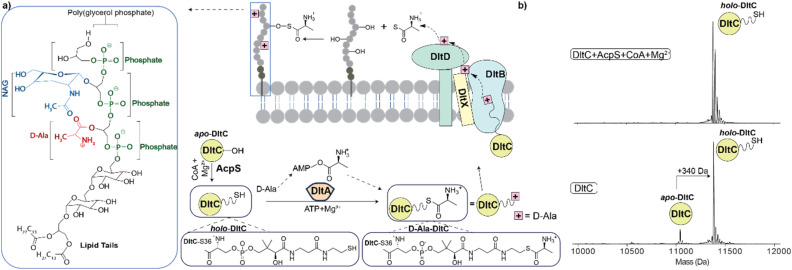
Dlt pathway and *in vitro* phosphopantetheinylation of DltC. (a) Schematic of LTA d-alanylation, including the chemical repeat unit of LTA and the d-Ala ester modification site. DltA activates d-alanine in an ATP-dependent reaction to form AMP-d-Ala, and the d-alanyl group is transferred to the phosphopantetheine thiol of *holo*-DltC. DltC then functions with downstream Dlt components, including DltB, DltX and DltD, to support d-Ala transfer to LTA. (b) Deconvoluted native mass spectra of purified DltC showing *apo*- and partially *holo*-DltC before AcpS treatment (bottom panel) and conversion to *holo*-DltC after incubation with AcpS, CoA and Mg^2+^ (top panel).

Central to d-alanylation is the *dlt* operon, in which DltA is an ATP-dependent d-alanine-d-alanyl carrier protein (Dcp) ligase^7^ that activates d-alanine through adenylation and subsequently transfers the d-alanyl group to the carrier protein DltC (Fig. 1a). The *apo*-form of DltC is modified with a phosphopantetheine (P-pant) moiety derived from coenzyme A (CoA) by the acyl carrier protein synthase AcpS.^8^ The resulting P-pant-DltC *holo*-form is then converted to d-alanyl-P-pant-DltC through the formation of a thioester bond between the d-alanyl group and the P-pant prosthetic group.^9–11^d-Ala from DltC is then transferred by the MBOAT protein DltB.^12,13^ Recent studies suggest that DltB transfers d-Ala to the conserved tyrosine of a small membrane protein, DltX, which then acts as a shuttle to deliver the d-Ala to DltD, a serine hydrolase that catalyses the final transfer to LTA.^7,9,11^ Because of its role in bacterial virulence, the Dlt pathway has emerged as a potential drug target, and several small molecules, including amsacrine derivatives and DBI-1, have been reported to inhibit Dlt function in *S. aureus* and *B. subtilis*.^13–15^

While DltA functions specifically to activate d-alanine for its incorporation into LTAs, it belongs to the broader adenylate-forming superfamily and shares a conserved catalytic domain architecture with the adenylation (A) domains of Nonribosomal Peptide Synthetases (NRPSs).^16,17^ High-resolution crystal structures of DltA (PDB: 3FCC, 3FCE),^18^ including complexes with substrates (d-Ala, ATP, Mg^2+^), and intermediates (d-Ala-AMP), have provided snapshots of the conserved two-step catalytic mechanism.^19^ These structures capture states analogous to adenylation and thioesterification in NRPS A-domains:Adenylation (AA + ATP → AA-AMP + PPi)Thioesterification (AA-AMP + Ppant → AA-S-Ppant + AMP)

Together, structural and biochemical studies have established the core chemistry of DltA catalysis and identified key interactions involved in substrate recognition and positioning.^20,21^ However, these approaches provide limited information on the distribution of DltA- and DltC-associated species present under defined solution-phase reaction conditions. In particular, DltA-bound adenylate, *holo*-DltC, d-Ala-loaded DltC, inhibitor-bound complexes, and off-pathway carrier-protein species can be difficult to distinguish using ensemble biochemical assays alone. This limits direct assessment of how substrate activation, carrier-protein loading, and inhibition are reflected in the composition of the reconstituted system. As a result, the mechanistic basis of inhibition within the Dlt pathway remains incompletely understood.

In this study, we used native mass spectrometry (MS) to monitor the enzymatic activities of DltA, DltC and AcpS under conditions that preserve protein complexes and carrier-protein modifications. Building on the established chemistry of the DltA/DltC reaction, we asked whether native MS could resolve the distinct reaction species formed during d-alanine activation and transfer. Specifically, we monitored ATP-dependent AMP-d-Ala formation by DltA, d-Ala transfer to *holo*-DltC, inhibition by a sulfamoyl-adenosine analogue, and the effects of mutations in the conserved ATP-binding P-loop of DltA. Our results show that native MS can distinguish adenylate formation, inhibitor binding and carrier-protein loading, while also detecting oxidation-associated *holo*-DltC species that affect *in vitro* reconstitution. Together, these findings establish native MS as a complementary approach for analysing phosphopantetheinyl-dependent carrier protein chemistry.

## Results

### Purified DltC is partially phosphopantetheinylated and can be fully converted to *holo*-DltC by AcpS

To study the d-alanylation process *in vitro*, we recombinantly expressed *B. subtilis* DltA and DltC in *E. coli* and purified both proteins using standard affinity chromatography protocols. Consistent with previous studies, both proteins were readily purified from soluble fractions to high purity.^21–23^ Interestingly, we were also able to purify DltA and DltC from membrane fractions to similar purity (Fig. S1). These results suggest that the DltA and DltC enzymes can anchor to or associate with membranes and likely carry out their functions near the membrane. For subsequent *in vitro* experiments, we used proteins purified from the soluble fraction to avoid complications arising from detergents.

We next analysed purified DltA and DltC using native MS. The resulting spectrum for DltA displays a single charge-state distribution that closely matches the anticipated mass of 57 949 ± 1.22 Da (Fig. S2). In contrast, the spectrum of DltC revealed two distinct charge-state series assigned to monomers and dimers in solution (Fig. S3). The peaks corresponding to monomeric forms have adduct peaks, suggesting a possible modification. The mass shift (∼340 Da) corresponds to the addition of a phosphopantetheinyl (P-pant) group, consistent with partial phosphopantetheinylation, likely due to endogenous AcpS activity in *E. coli*.^22^ To confirm the presence of the P-pant modification, we performed a P-pant ejection assay under denaturing MS conditions. This analysis detected a characteristic fragment ion at *m*/*z* 261.1, consistent with ejection of the P-pant moiety from *holo*-DltC (Fig. S4).^23^ The measured masses for *apo*- and *holo*-DltC, and the DltC dimer, are 11 043 ± 0.2 Da, 11 380 ± 0.32 Da, and 22 760 ± 0.42 Da, respectively (Fig. 1b). Notably, we did not detect a corresponding *apo*-DltC dimer under these conditions. Because dimerisation was observed only for the phosphopantetheinylated form, most likely arising from oxidation of the phosphopantetheine thiol to form a disulfide-linked *holo*-DltC dimer. Consistent with this assignment, treatment of purified DltC with DTT reduced the abundance of the dimer and increased the population of monomeric DltC in denaturing MS spectra (Fig. S5). Similar oxidation-associated dimerisation has been reported previously for carrier proteins, including DltC.^24–26^

To generate homogeneous *holo*-DltC, we performed *in vitro* phosphopantetheinylation using purified *Staphylococcus aureus* AcpS.^27^ DltC and AcpS (5 µM each) were incubated with coenzyme A (18 µM) in 200 mM ammonium acetate, pH 8.0, for 30 min on ice before native MS analysis. The resulting spectra showed a single charge-state series in the monomer region corresponding exclusively to *holo*-DltC. A uniform mass increase of ∼340 Da relative to the *apo* form confirmed complete conversion to the *holo* state (Fig. 1b). These results show that AcpS efficiently phosphopantetheinylates DltC under solution conditions compatible with native MS. The resulting *holo*-DltC was used in all downstream transfer assays.

### Direct observation of AMP-d-Ala intermediate formation by DltA

**Fig. 2:**
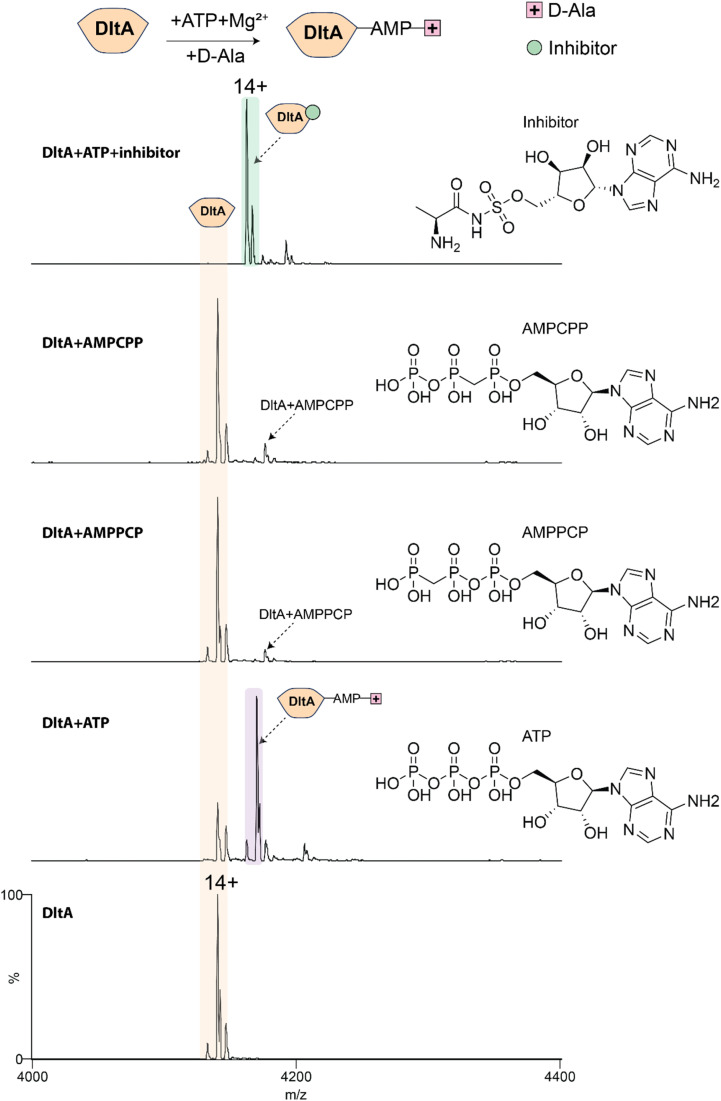
Monitoring DltA adenylation activity and inhibition. Native mass spectra of DltA in the absence and presence of ATP, other nucleotides and inhibitor. Note that d-Ala and Mg^2+^are included in all the reactions. AMP-d-Ala formation was detected only in the presence of DltA, ATP, Mg^2+^ and d-Ala. Non-hydrolysable ATP analogues supported weak binding but not detectable AMP-d-Ala formation. The sulfamoyl-adenosine inhibitor bound strongly to DltA and prevented detectable AMP-d-Ala formation.

We next examined the activity of purified DltA. DltA activates d-alanine in an ATP-dependent manner through the formation of a high-energy AMP-d-Ala adenylate intermediate; the d-Ala group is then transferred to the Ppant thiol of *holo*-DltC.^11^ To monitor the formation of the AMP-d-Ala intermediate, we mixed 4 µM DltA with 40 µM ATP, 20 µM MgCl_2_, and 2 mM d-Ala and analysed the sample by native MS. The spectrum revealed clear adduct peaks at ∼418 Da, consistent with the covalent binding of AMP-d-Ala to form an intermediate (Fig. 2). This mass difference matched the expected addition of AMP and d-alanine, confirming successful adenylation. This intermediate was absent in reactions lacking ATP or d-Ala, confirming strict substrate dependence for adenylation (Fig. S6).

Having observed AMP-d-Ala intermediate formation, we explored the possibility that DltA could also form a similar adduct with l-Ala. We performed analogous experiments as above, using l-Ala instead of d-Ala, and acquired MS data (Fig. S7). In this case, we detected the addition of ATP, but no evidence for the AMP-l-Ala intermediate. Consistent with previous biochemical studies of DltA stereoselectivity,^28,29^ this result confirms that l-Ala is not adenylated under our assay conditions.

To further probe the specificity of DltA for nucleotide substrates, we tested whether other ATP variants, namely AMPPCP and AMPCPP, could support adenylation. We performed native MS analyses of these compounds using the method described above for ATP. The data surprisingly reveal only weak noncovalent binding between these compounds, with no evidence of AMP-d-Ala intermediate formation (Fig. 2). These data indicate that detectable adenylation requires ATP.

### A sulfamoyl-adenosine inhibitor blocks detectable DltA adenylation

Recent studies have identified the inhibitor (5′-*O*-[*N*-(d-alanyl)-sulfamoyl]adenosine), which blocks DltA-catalysed transfer of d-alanine to DltC *in vitro*.^30–32^ We reasoned that this inhibitor could bind directly to *B. subtilis* DltA and inhibit adenylation activity. When DltA (4 µM) was incubated with the inhibitor (40 µM) and analysed by native MS, we observed a species consistent with inhibitor binding and little *apo* protein remaining (Fig. S8). We also observed species with up to two inhibitor molecules bound. When comparing this result with that of ATP binding, we found that DltA binds the inhibitor with a much higher affinity than ATP (Fig. S6). This tighter binding is consistent with previous studies of acyl-sulfamoyladenosine inhibitors, which mimic the acyl-AMP reaction intermediate and often inhibit adenylation domains with low-nanomolar to sub-micromolar potency. For example, d-alanyl-AMS was previously shown to inhibit *B. subtilis* DltA with a Ki of 232 nM,^30^ while related acyl-AMS inhibitors targeting other adenylation enzymes, including salicyl-AMS against MbtA and aminoacyl-AMS derivatives against NRPS adenylation domains, have been reported to inhibit with low-nanomolar to sub-micromolar potency.^33–36^ Thus, the high-affinity interaction observed here by native MS is consistent with the established tight-binding behaviour of AMS-based adenylation-domain inhibitors.

We then tested the effect of this inhibitor on the adenylation process by incubating DltA (4 µM) with 40 µM ATP, 20 µM MgCl_2_, 2 mM d-Ala and 40 µM inhibitor. Native mass spectra show exclusive binding of the inhibitor, with no peaks corresponding to the AMP-d-Ala intermediate (Fig. 2). These data indicate that the inhibitor occupies the active site and prevents DltA from forming the adenylate intermediate.

### Mutations in the P-loop motif of DltA alter adenylate formation

**Fig. 3:**
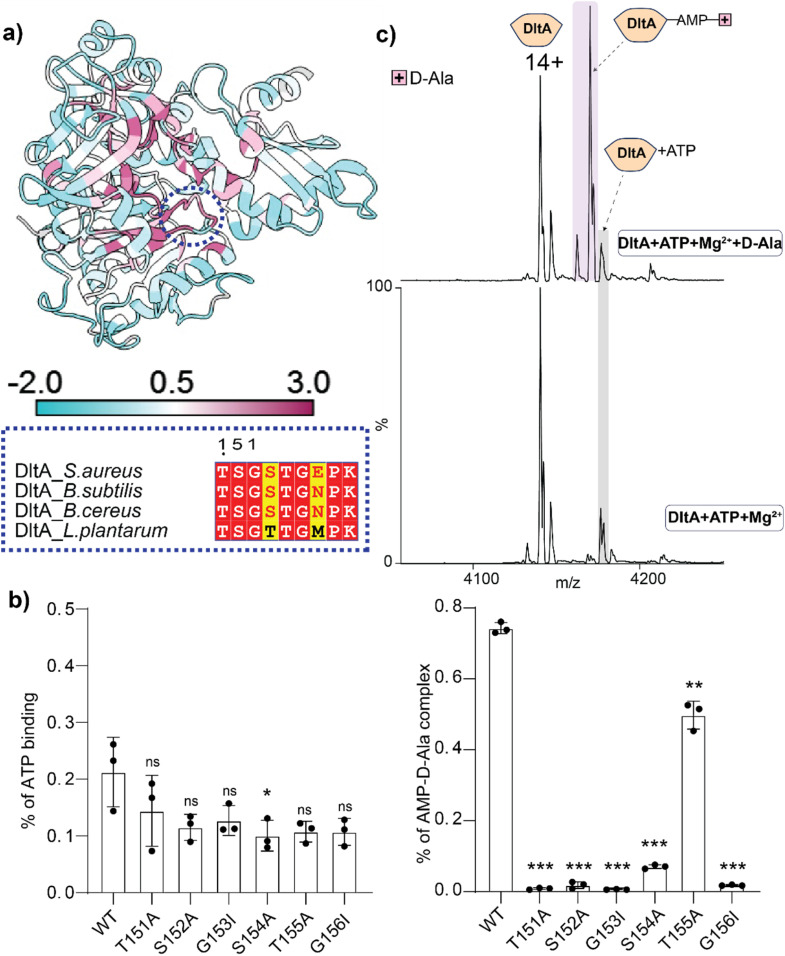
P-loop residues of DltA influence ATP binding and AMP-d-Ala formation. (a) Conservation analysis of *B. subtilis* DltA (PDB: 3E7W).^27^ The conserved P-loop region is highlighted in blue, and sequences for this region are shown for four species. (b) Quantification of ATP-bound species by native MS. (c) Mass spectrometry data showing the formation of the AMP-d-Ala intermediate (top spectrum) with ATP as control (bottom spectrum). Bar chart showing the amount of intermediate formed in each case in comparison with each mutant and the WT. For statistics, *n* = 3, *p* < 0.05 = *, *p* < 0.01 = **, *p* < 0.001 = ***.

Motivated by the inhibitory effect of the sulfamoyl-adenosine compound, we next examined whether mutations in the conserved ATP-binding P-loop could perturb DltA function. We focused on the conserved ATP-binding P-loop motif residues (151–159) to assess their importance in adenylation (Fig. 3 and S9). Because this motif is known to contribute to ATP binding and adenylate formation in adenylate-forming enzymes, activity disruption was expected for strongly perturbing mutations.^11,37^ We generated plasmids encoding T151A, S152A, G153I, S154A, T155A, and G156I, and purified the respective proteins using the methods optimised above for the wild type (Fig. S10). These proteins were then subjected to native MS. The data show that all proteins were purified to similar quality (Fig. S11), except for G153I and S154A. We tested the effect of these mutations on ATP binding using native MS. The data show reduced affinity for all mutants (Fig. S12), consistent with their predicted role in ATP binding.

We next explored whether the weakened ATP binding in the mutants correlated with their adenylation activity. We acquired native MS data for each mutant in the presence of ATP and d-ala, as for the wild type, and measured the amount of the AMP-d-ala intermediate formed. Intermediate formation was almost completely abolished in T151A, S152A, G153I and G156I, whereas S154A and T155A retained detectable activity. Among all variants tested, T155A showed the highest residual activity, although this remained below wild-type levels (Fig. 3b, c and S13). These data highlight the importance of the conserved P-loop residues in DltA catalysis.

### *Holo*-DltC accepts d-alanine from DltA

**Fig. 4:**
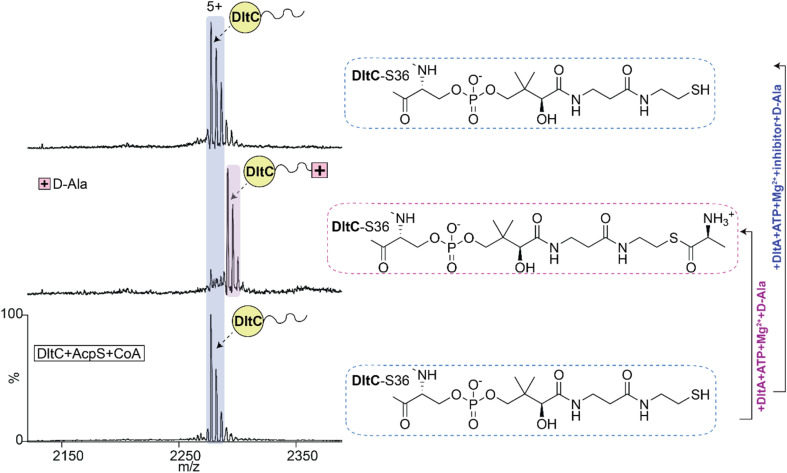
Establishing the methodology for tracking the transfer of d-alanine to DltC. Monitoring transfer of d-alanine to DltC (middle spectrum) and its inhibition following the addition of the inhibitor (top spectrum). The spectrum with the inhibitor (top) mimics that of *holo*-DltC (bottom).

Having established the complete *in vitro* phosphopantetheinylation of DltC and directly observed the formation of the AMP-d-Ala intermediate by DltA, we next asked whether d-alanine transfer to *holo*-DltC could be detected by native MS. We incubated the DltA adenylation reaction mixture (3 µM DltA, 20 µM MgCl_2_, 40 µM ATP, and 2 mM d-alanine) with *holo*-DltC (9 µM) and analysed the products by native MS. A new DltC species with a mass increase of ∼70 Da was observed, consistent with d-Ala thioester formation on the P-pant arm (Fig. 4 and S14). This loading reaction required both *holo*-DltC and active DltA and was strongly reduced or absent when the oxidation-associated *holo*-DltC dimer was present. These results are consistent with the established requirement for a reduced phosphopantetheine thiol in productive d-Ala transfer.

### Inhibitor binding and P-loop mutations reduce d-Ala transfer to DltC

We next repeated the d-Ala transfer assay in the presence of the inhibitor. As expected from the adenylation assay, no transfer of the d-Ala moiety to DltC was detected (Fig. 4). This establishes a direct native MS link among inhibitor-bound DltA, the loss of detectable adenylate formation, and the failure of carrier-protein loading.

**Fig. 5:**
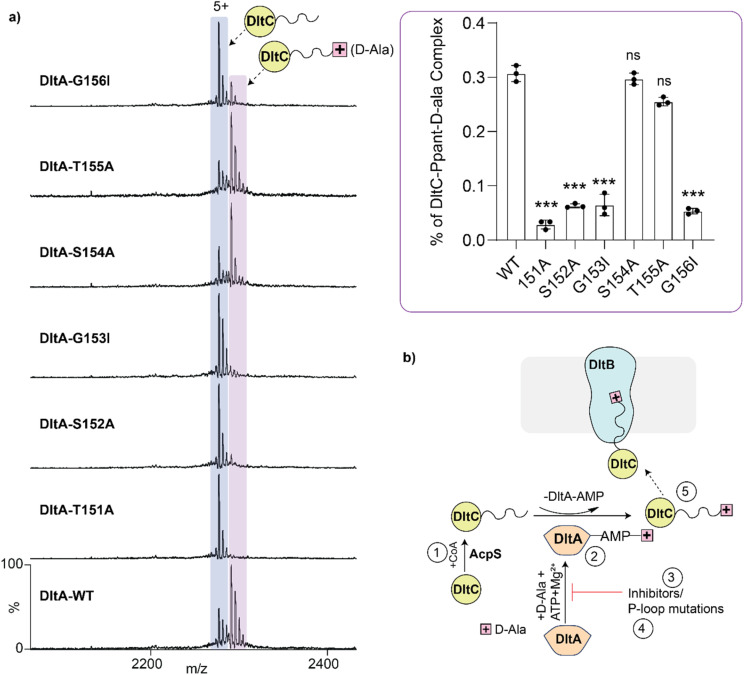
Effects of DltA P-loop variants on d-Ala transfer to DltC. (a) Native MS spectra and quantification of d-Ala-loaded *holo*-DltC formed in reactions containing wild-type DltA or P-loop variants. d-Ala transfer was readily detected in wild-type DltA and retained to varying extents in S154A and T155A. Inset, bar chart of percentage DltC-Ppant-d-Ala complex observed in each case. For statistics, *n* = 3, *p* < 0.001 = ***, *p* < 0.01 = **. (b) Summary schematic highlighting the key steps monitored in this study.

We also tested the P-loop mutants in the transfer assay and quantified formation of DltC–Ppant–d-Ala from the spectra. T151A, S152A, G153I and G156I showed low transfer activity, consistent with their reduced AMP-d-Ala formation. T155A retained substantial transfer activity, in line with its detectable adenylate formation. Interestingly, S154A supported relatively efficient d-Ala transfer despite low detectable AMP-d-Ala occupancy in the DltA-only assay (Fig. 5a). Overall, these data confirm that adenylation by DltA is required for subsequent d-Ala loading onto DltC and show that native MS can directly track both enzymatic steps and their disruption.

## Discussion

This study establishes native MS as a direct, label-free approach for monitoring the early cytosolic steps of the d-alanylation pathway. DltA and DltC coordinate d-alanine activation and carrier-protein transfer before downstream incorporation into LTA. Although the core chemistry of d-Ala adenylation, stereoselective substrate activation, DltC loading and sulfamoyl-adenosine inhibition has been established previously,^9,11,22,27,28,30^ our *in vitro* reconstitution shows that these events can be followed directly and in parallel under solution conditions compatible with protein complexes and carrier-protein modifications. Native MS resolved *apo*- and *holo*-DltC, DltA-bound AMP-d-Ala, inhibitor-bound DltA and d-Ala-loaded *holo*-DltC, thereby allowing productive intermediates, inhibitor-bound states, and carrier-protein loading to be analysed in the same set of experiments.

We detected DltA and DltC in membrane-associated fractions, consistent with the possibility that a subset of these proteins may have some degree of membrane association *in vivo*. This interpretation is in line with previous structural and biochemical studies showing that DltC interacts with the membrane-embedded protein DltB, supporting a model in which d-Ala transfer is spatially organised within the Dlt pathway.^12,13,22^ Native MS also revealed oxidation-associated *holo*-DltC dimers. Because dimer formation was restricted to Ppant-modified DltC and was reduced under reducing conditions, we interpret this species as an off-pathway, disulphide-linked product formed *via* the Ppant thiols during purification or handling, rather than as a regulatory state.^24,25^ Its inability to support d-Ala loading is consistent with the loss of the free Ppant thiol required for thioester formation. This observation highlights an important practical consideration for reconstituting Ppant-dependent chemistry: the redox state of carrier proteins must be controlled to preserve the transfer-competent *holo* form.^38^

Direct detection of DltA-bound AMP-d-Ala required ATP, Mg^2+^ and d-Ala. No equivalent intermediate was observed with l-Ala or with non-hydrolysable ATP analogues, consistent with previous biochemical studies showing ATP-dependent activation of d-alanine by DltA from *B. subtilis* and *Lactobacillus casei*, which reported activation only in the presence of d-alanine.^28–30^ A sulfamoyl-adenosine inhibitor that mimics the adenylate intermediate bound strongly to DltA and blocked detectable AMP-d-Ala formation, thereby preventing downstream transfer to *holo*-DltC. These results provide orthogonal support for the proposed adenylate-mimicry mechanism and are consistent with previous studies showing that inhibition of DltA can sensitise Gram-positive bacteria to host antimicrobials by disrupting DltA-dependent resistance mechanisms.^30,39^ More broadly, this observation places DltA within a well-established inhibitor paradigm for adenylate-forming enzymes, in which acyl-sulfamoyladenosines act as non-hydrolysable mimics of the acyl-AMP intermediate and often bind with high affinity. Related AMS-based inhibitors have been reported to inhibit diverse adenylation enzymes, including NRPS adenylation domains and siderophore-biosynthetic adenylating enzymes, with low-nanomolar to sub-micromolar potency.^33–36^ Thus, the strong binding and inhibitory effect observed here further support adenylate mimicry as a useful strategy for probing and potentially targeting DltA function.^33^

Mutations in the conserved DltA P-loop further showed that native MS can distinguish nucleotide binding, adenylate formation, and carrier-protein transfer. Variants T151A, S152A, G153I and G156I showed markedly impaired AMP-d-Ala formation, whereas S154A and T155A retained detectable activity, with T155A approaching wild-type levels. Transfer assays revealed an approximately 70 Da mass shift on *holo*-DltC, consistent with d-Ala thioester formation. This transfer was blocked by the inhibitor and by disruptive P-loop mutations. Notably, S154A showed low, detectable AMP-d-Ala occupancy but supported efficient d-Ala transfer to DltC, suggesting that low steady-state adenylate levels may be sufficient for thioester formation or that this mutation alters partitioning between adenylation and transfer. Further kinetic analysis will be required to distinguish these possibilities.

Together, our findings support the established model in which DltA activates d-alanine and *holo*-DltC serves as its carrier for delivery to LTA.^40^ More specifically, they show that native MS can distinguish productive reaction intermediates, inhibitor-bound states, and off-pathway carrier-protein oxidation states in a single set of experiments. This capability should be useful not only for the Dlt pathway but also for other systems that rely on phosphopantetheinylated carrier proteins, including NRPSs, polyketide synthases, and fatty acid synthases. Although the present study does not define the DltA–DltC binding interface, future work combining native MS with structure-guided interface mutagenesis, kinetic analysis, and structural modelling should help reveal how DltA engages DltC and how carrier-protein transfer is coordinated with downstream membrane-associated Dlt components.

## Methods and materials

### Plasmid constructions

Gene blocks of full-length *B. subtilis* DltA and DltC, and *S. aureus* AcpS were purchased from Integrated DNA Technologies Inc., Leuven, Belgium. All genes were inserted into a modified pET15b vector between Ndel and Nhel endonuclease restriction sites by In-Fusion HD cloning kit (Clontech), with the addition of a C-terminal 6xHis tag after a TEV cleavage site. Primers of DltA mutants were constructed (Table S1) and ordered from Integrated DNA Technologies Inc. Plasmids for DltA, DltC, AcpS and DltA mutants were transformed by heat-shock into Stellar™ competent cells (Clontech), and colonies were selected for plasmid extractions using a GeneJET Plasmid Miniprep Kit (Thermo Scientific). The DNA sequences of the plasmids were verified by sequencing (Source Bioscience).

### Protein expression and purification

Plasmids were used to transform chemically in *E. coli* BL21 (DE3) and selected on LB/agar containing 100 µg mL^−1^ ampicillin at 37 °C. Single colonies were used to inoculate 100 mL LB containing 100 µg mL^−1^ ampicillin and were grown at 37 °C overnight for 16 h. For large-scale protein expression, 10 mL of the overnight culture was aseptically transferred into 1 L of LB/ampicillin (100 µg mL^−1^). Cells were allowed to grow at 37 °C for 3–4 h until the OD_600_ nm reached 0.6–0.8. The expression was induced with isopropyl-β-d-thiogalactopyranoside (IPTG) at a final concentration of 0.5 mM for 4 h at 37 °C. Cells were harvested by centrifugation at 5000×*g* for 10 min at 4 °C, pellets were resuspended in resuspension buffer (20 mM Tris pH 8.0, 200 mM NaCl) and lysed immediately or NO_2_ snap frozen and stored at −80 °C until required.

Cells were thawed on ice and resuspended in a buffer (20 mM Tris pH 8.0, 150 mM NaCl) containing lysozyme and protease-inhibitor cocktail tablets and then lysed using Sonication (total processing time = 10 min, pulse-on : pulse-off = 3 : 6, amplitude = 65 with a Mili-tip). The cell lysate was centrifuged (20 000 g for 20 minutes at 4 °C) to separate the protein from the crude material. The supernatant was filtered using a 0.45 µm filter syringe and loaded onto a 5 mL HisTrap column with Ni-NTA resin beads pre-equilibrated with 20 mM Tris pH 8.0, 150 mM NaCl, 20 mM imidazole. 30 mL of buffer A was loaded onto the column, followed by 30 mL 20 mM Tris pH 8.0, 150 mM NaCl, 80 mM imidazole to elute common impurities. Finally, the target proteins were eluted with 30 mL of 20 mM Tris, pH 8.0, 150 mM NaCl, and 250 mM imidazole and dialysed overnight against a 20 mM Tris, pH 8.0, 150 mM NaCl, 10% v/v glycerol buffer to remove imidazole. After the removal of imidazole, the target proteins were concentrated and snap-frozen until further use.

### Native mass spectrometry

Proteins were buffer-exchanged into 350 mM ammonium acetate (pH 8.0) using BioSpin-6 (Biorad) columns. About 3 µL protein sample was introduced directly into the mass spectrometer (Q-Exactive UHMR mass spectrometer, Thermo Fisher Scientific) using a gold-coated borosilicate capillary (prepared in-house, originally from Harvard Apparatus). The instrument parameters were as follows: capillary voltage 1.0 kV, S-lens RF 100%, quadrupole selection from 1000 to 20 000 *m*/*z* range, collisional activation in the HCD cell 50–100 V, trapping gas pressure setting 7.5, temperature 200 °C, and resolution of the instrument 12 500. The noise level was set at 3 rather than the default value of 4.64. No in-source dissociation was applied. Data were analysed using Xcalibur 4.2 (Thermo Scientific) and UniDec software packages. All experiments were repeated with similar outcomes. Statistical analysis was performed using GraphPad Prism, comparisons for two groups (with one as a control) were calculated using Dunnett's test (One-Way ANOVA). Exact *n* and *P* values are indicated in figure legends.

### Conservation analysis

Conservation analysis was performed using ConSurf on UniProt database structures of *B. subtilis* DltA. Homologues were identified using HMMER to search UniRef90 with an *E*-value threshold of 0.0001, requiring sequence identity between 35% and 95%. MAFFT generated the multiple sequence alignments (MSAs), from which 150 sequences were selected for Bayesian conservation score calculation.

## Author contributions

Conceptualisation of project: Y. W., and J. R. B.; methodology: Y. W., and J. R. B.; investigation: Y. W., H. S., M. A. A., T. J. E., and J. R. B.; funding acquisition: C. V. R., and J. R. B.; project administration: J. R. B.; supervision: J. R. B.; writing – original draft: Y. W., and J. R. B.; writing – review and editing: Y. W., J. P. G., T. J. E., R. C. M., C. V. R., and J. R. B. All authors commented on the final version of the manuscript.

## Conflicts of interest

C. V. R. is founder and consultant of OMass Therapeutics. All other authors have no competing interests.

## Supplementary Material

RA-016-D6RA04213A-s001

## Data Availability

The raw mass spectrometry data that support the findings of this study have been deposited in the Figshare database (https://doi.org/10.6084/m9.figshare.32508129). Supplementary information (SI): tables and figures that are already described and referenced in the main text. See DOI: https://doi.org/10.1039/d6ra04213a.
