# Supplementary material for: Native mass spectrometry reveals DltA catalysis, DltC loading, and inhibition in the d-alanylation pathway
Source: RSC Adv. 2026 Jun 2;16(32):29834–43. doi: 10.1039/d6ra04213a (PMC13231372; doi:10.1039/d6ra04213a)
Supplement: RA-016-D6RA04213A-s001 [file RA-016-D6RA04213A-s001.pdf]

Supplementary Information (SI) for RSC Advances.
This journal is © The Royal Society of Chemistry 2026

Native mass spectrometry reveals DltA catalysis, DltC loading, and inhibition in the D-alanylation pathway

Yi Wang[1], Hannah Squires[1], Marian Aba Addo[2], Jospeh P Gerdt[2], Tarick J El-Baba[3,4], R Craig MacLean[5], Carol V Robinson[3,4], Jani R Bolla[1*]

[1]*Department of Biochemistry, University of Oxford, Oxford, OX1 3QU*

[2]*Department of Chemistry, Indiana University Bloomington IN 47405 USA*

[3]*Department of Chemistry, University of Oxford, Oxford, OX1 3QZ*

[4]*Kavli Institute for Nanoscience Discovery, University of Oxford, Oxford, OX1 3QU*

[5]*Department of Biology, University of Oxford, Oxford, OX1 3RB*

**Correspondence: Jani R Bolla (jani.bolla@bioch.ox.ac.uk)*

Running title: Native mass spectrometry reveals DltA-catalysed activation of D-alanine and DltC loading in the D-alanylation pathway

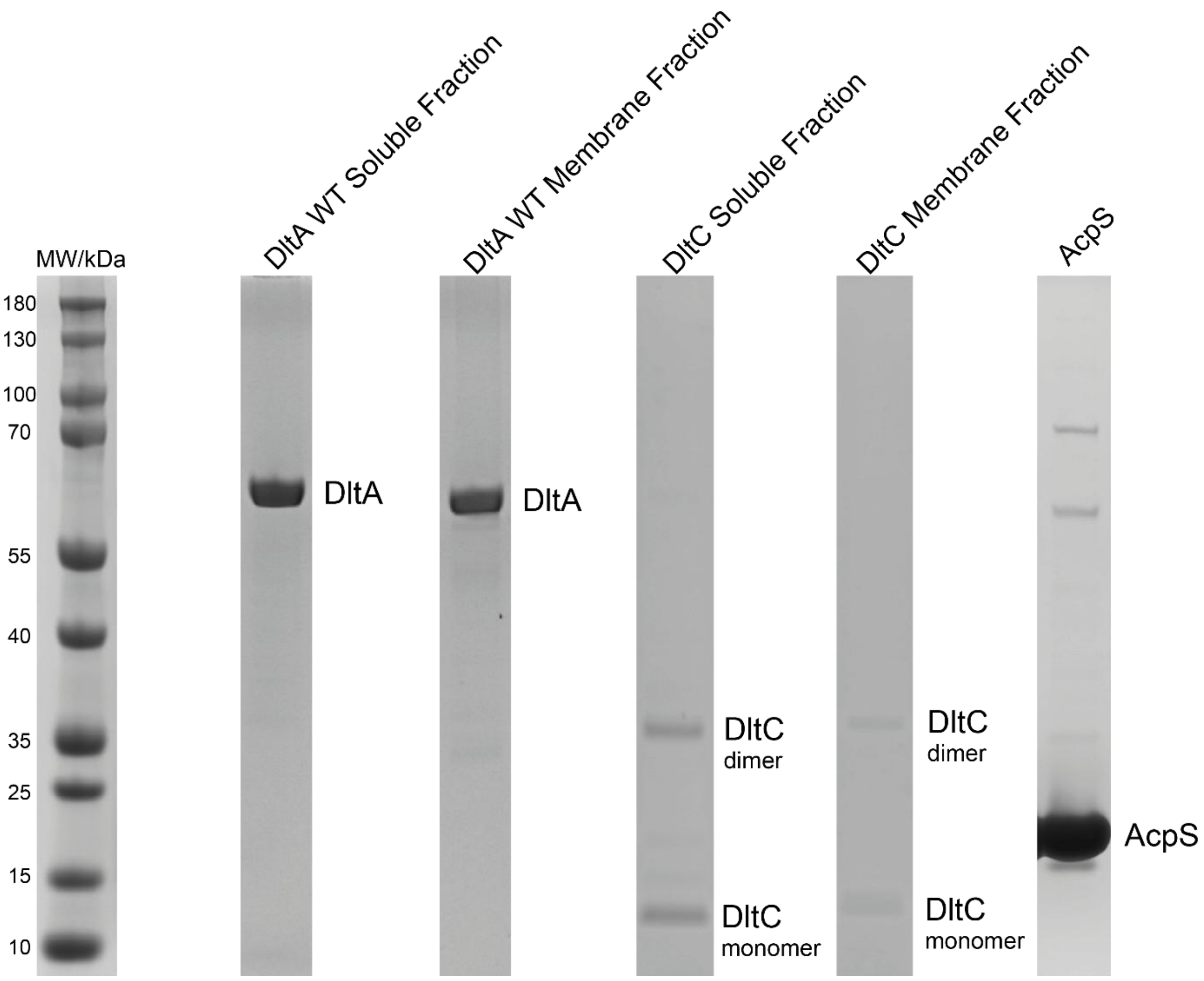

Figure S1: DltA and DltC can be purified from both soluble and membrane fractions. SDS-PAGE analyses of purified proteins. Both DltA and DltC can be purified from soluble and membrane fractions to similar purity levels. The gels were run under non-reducing conditions. All proteins show bands at their expected mass range. The ladder used here is the Precision Plus Protein Unstained Standards from BIO-RAD™.

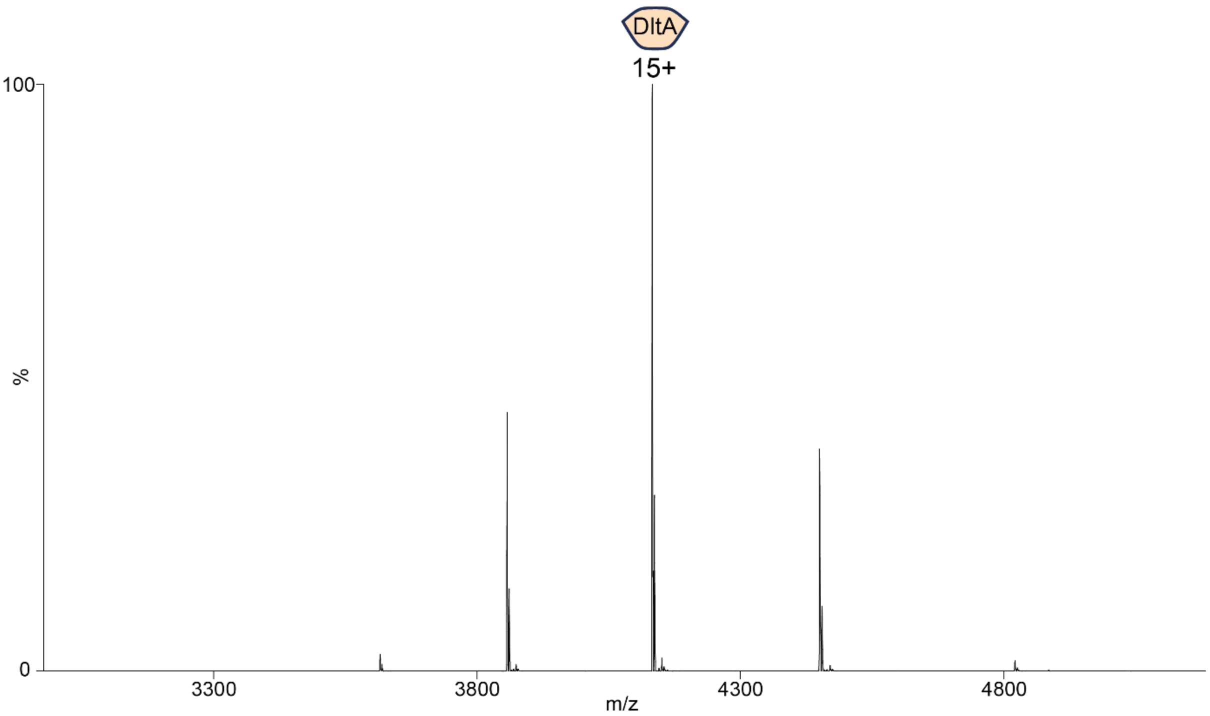

Figure S2: Native MS analysis of DltA. The protein was liberated from a buffer containing 200 mM ammonium acetate (pH 8.0) using a collisional activation of 50 V. The spectrum shows a charge state series whose mass corresponds to monomeric DltA. Theoretical and measured masses are listed in Table S2.

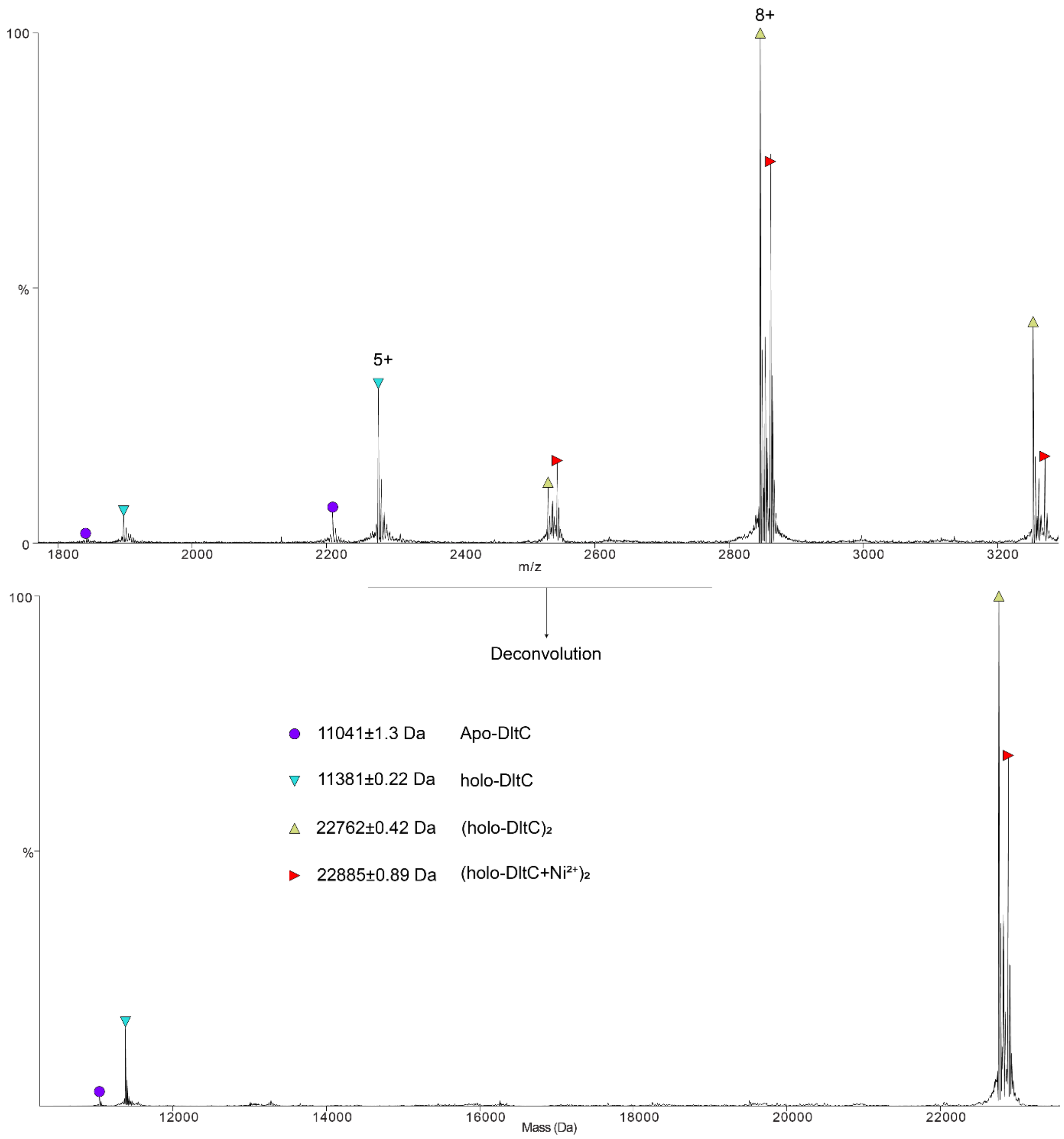

Figure S3: DltC is in monomer-dimer equilibrium. Native mass spectrum of purified DltC shows several charge state series corresponding to monomeric apo-DltC, holo-DltC and dimeric *holo*-DltC (top panel). Bottom panel show the deconvoluted masses.

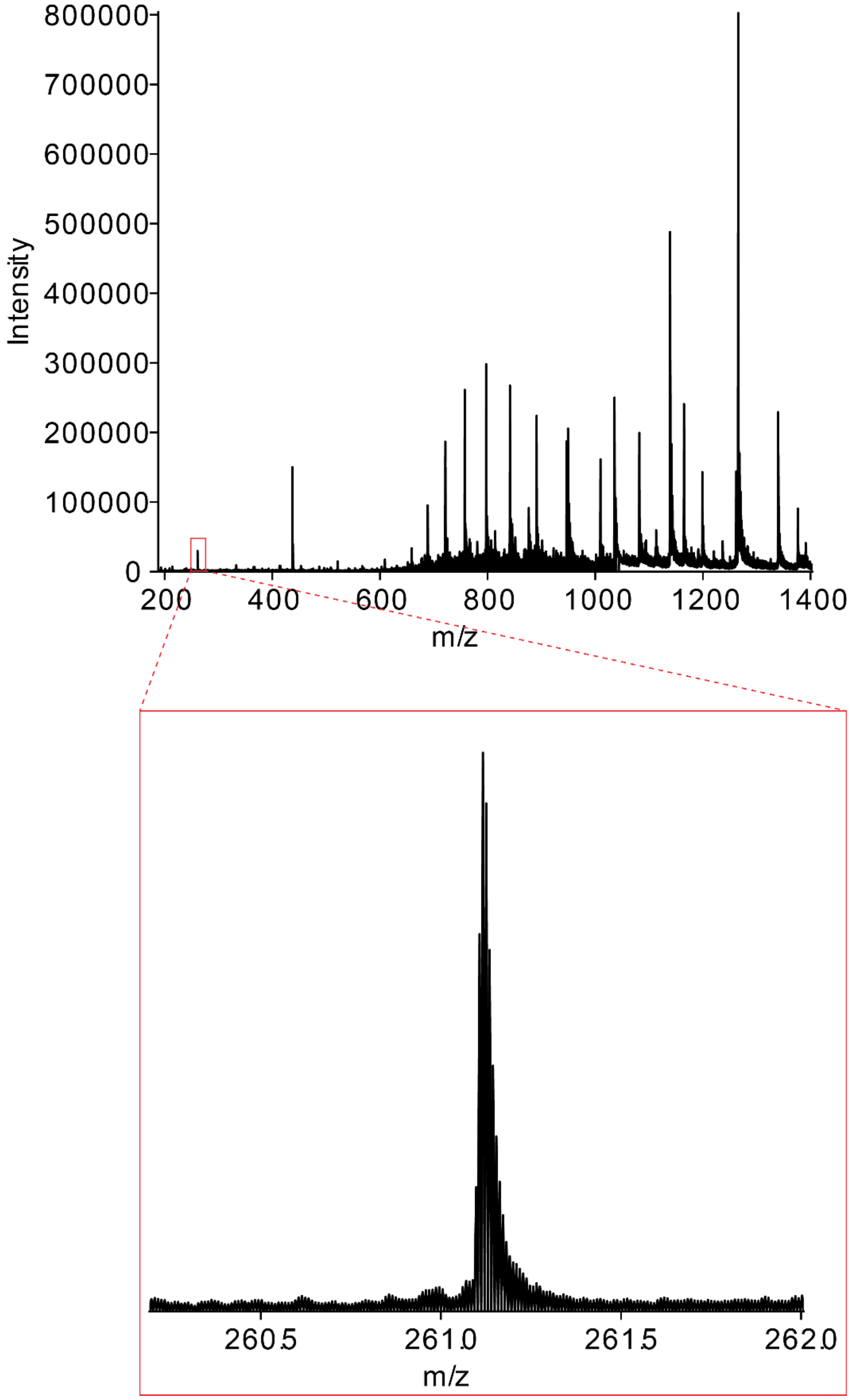

Figure S4: Detection of P-pant ejection from *holo*-DltC. Denaturing mass spectrometry of purified DltC shows a characteristic phosphopantetheine fragment ion at m/z 261.1, consistent with ejection of the P-pant moiety from *holo*-DltC.

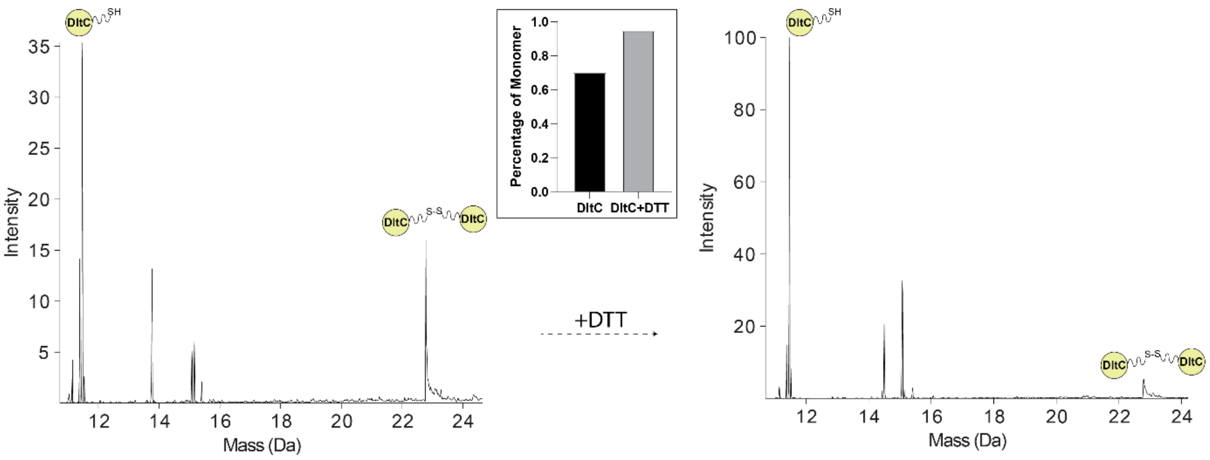

Figure S5: DTT disrupts the disulfide-linked *holo*-DltC dimer. Deconvoluted denaturing mass spectra of *holo*-DltC in the presence or absence of DTT show that DTT treatment increases the population of monomeric DltC.

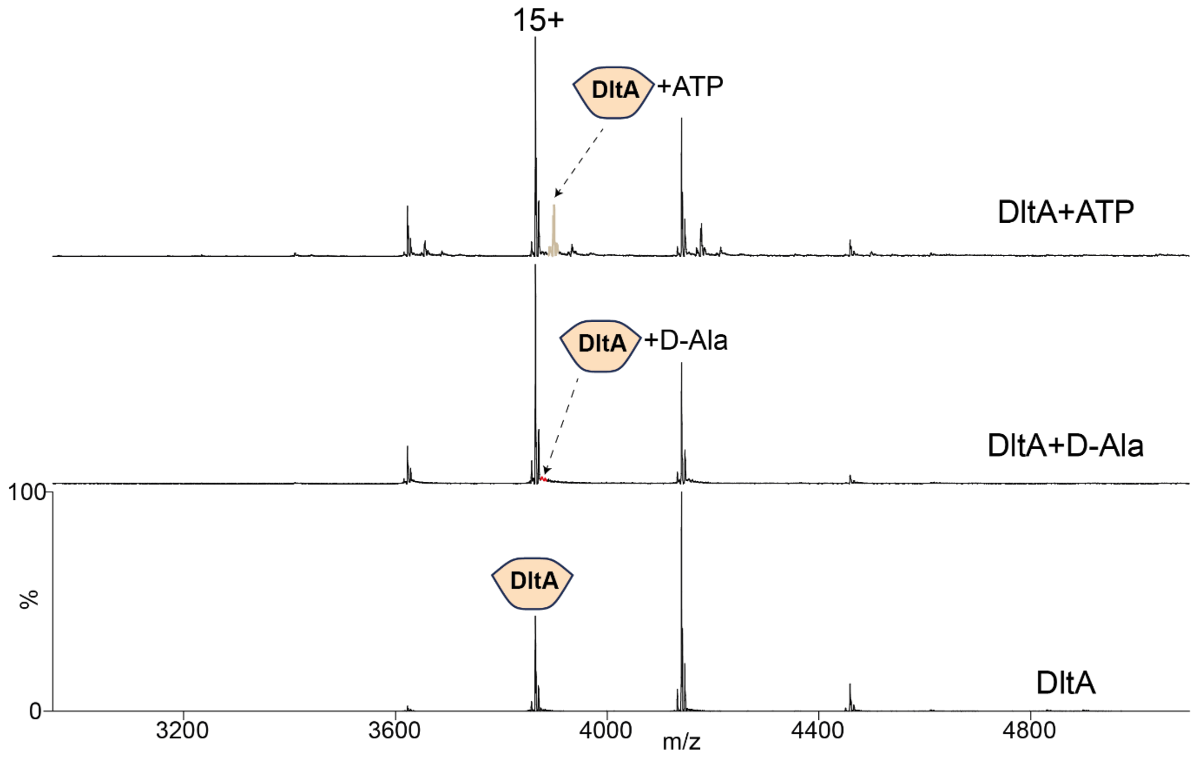

Figure S6: Binding of DltA with its substrates. Mass spectra of DltA in the absence (bottom) and presence of its substrates, D-ala (middle) and ATP (top). Adduct peaks are highlighted to show the non-covalent interaction between the protein and substrates. Theoretical and measured masses are listed in Table S3.

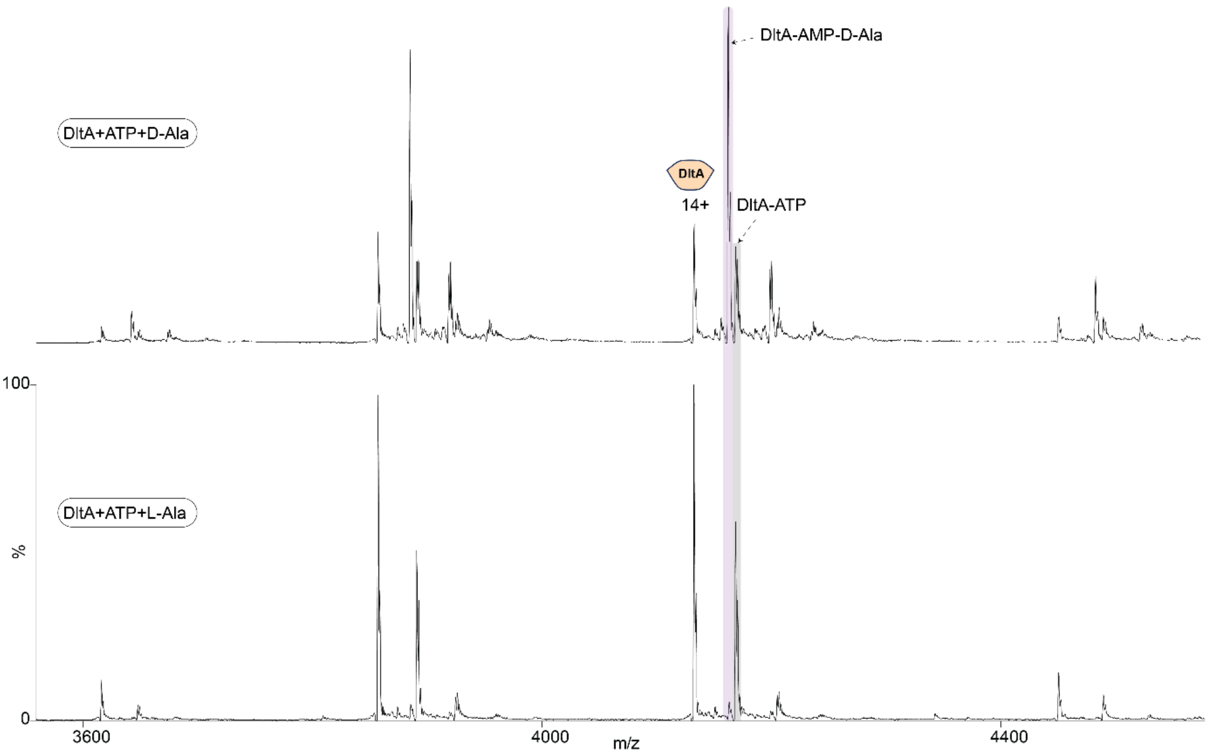

Figure S7: DltA prefers D-Ala over L-Ala for adenylation. Mass spectra of DltA in the presence of L-Ala (bottom) and D-Ala (top). Adduct peaks corresponding to successful adenylation can only be observed in the case of D-Ala (highlighted), but with little to no adenylation in the case of L-Ala. Theoretical and measured masses are listed in Table S3.

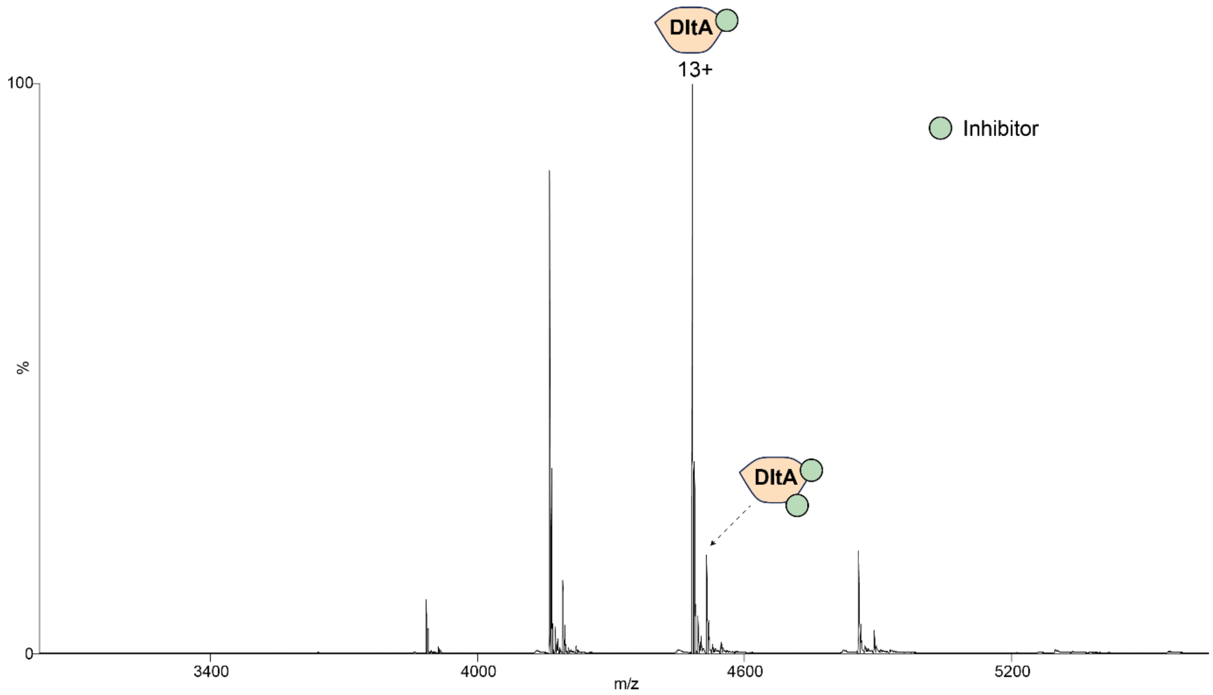

Figure S8: Inhibitor binds avidly to DltA. Mass spectrum of DltA with its inhibitor (5′-*O*-[*N*-(D-alanyl)-sulfamoyl]adenosine). The spectrum displays mostly protein bound to the inhibitor, with no *apo* protein, suggesting a strong affinity. Theoretical and measured masses are listed in Table S3.

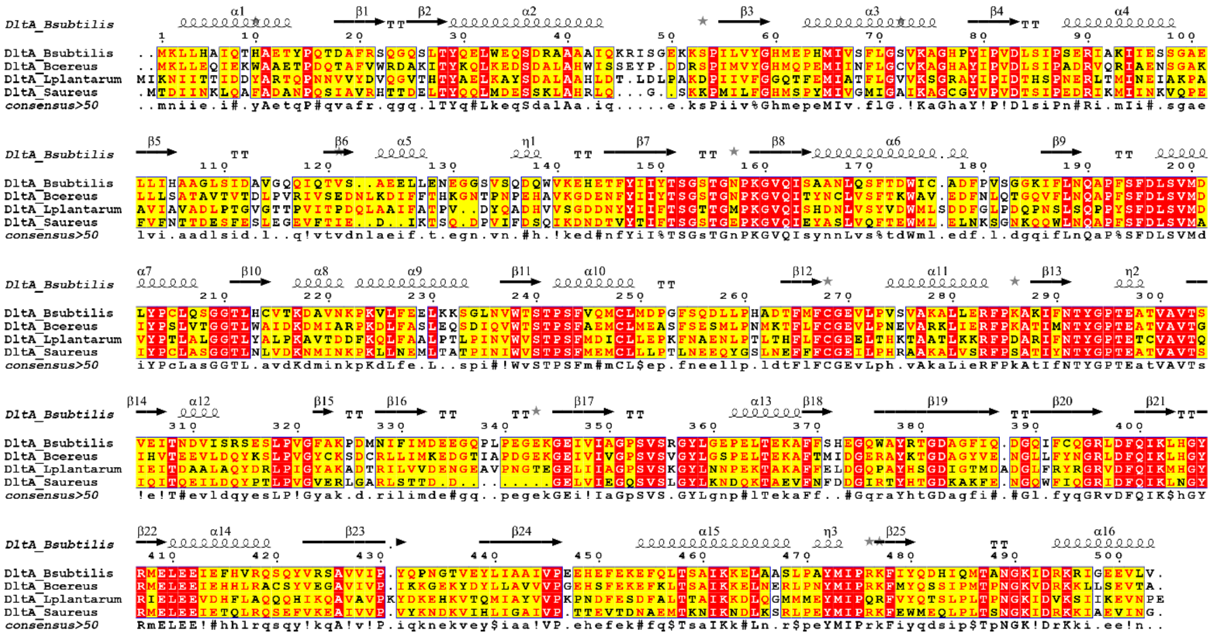

Figure S9: Multiple sequence alignment for *B. subtilis* DltA compared to other species. Sequence alignment of *B. subtilis* DltA with DltA from *Bacillus cereus* (*Bcereus*), *Lactobacillus plantarum* (*Lpantarum*), and *Staphylococcus aureus* (*Saureus*). Most of the conserved residues were located in the P-Loop region (Residue 151-160) and binding pockets (Residue 265-270 and 291-297).

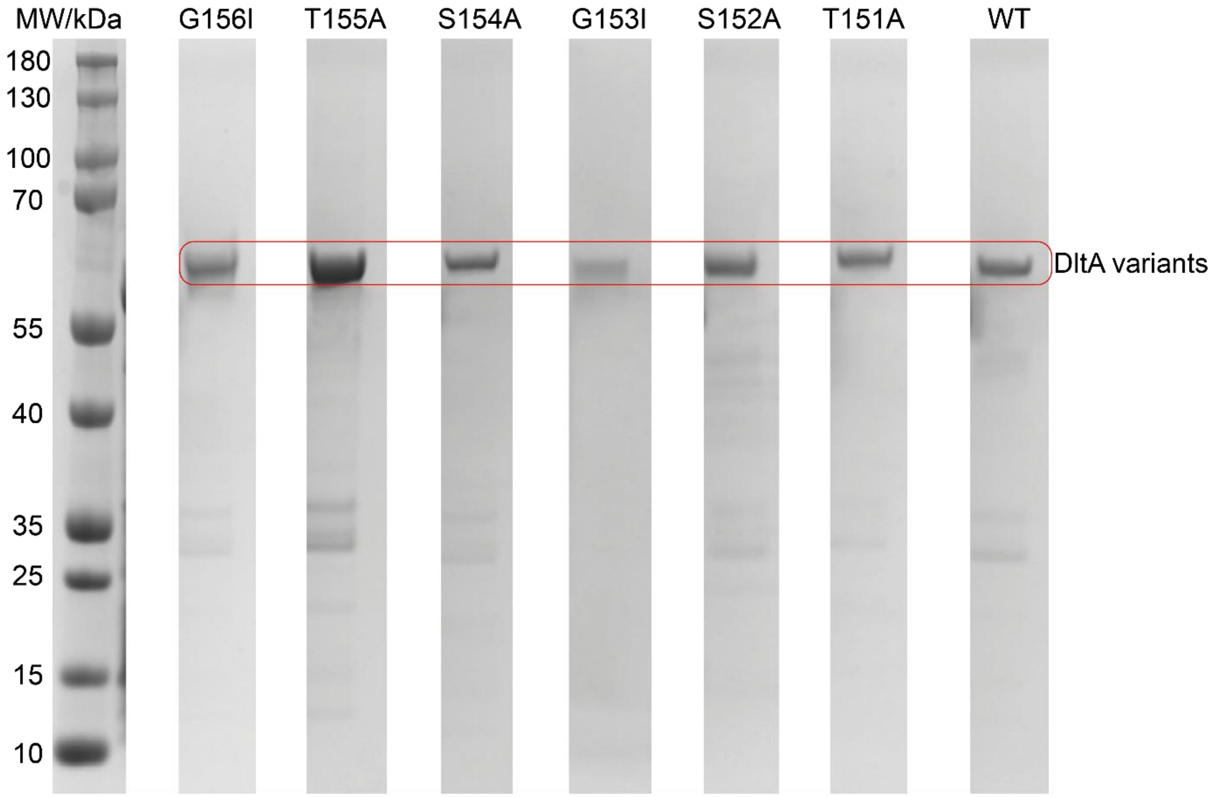

Figure S10: Purification of DltA P-loop mutants. SDS-PAGE analyses of purified DltA P-loop mutants show similar purity to the wild type.

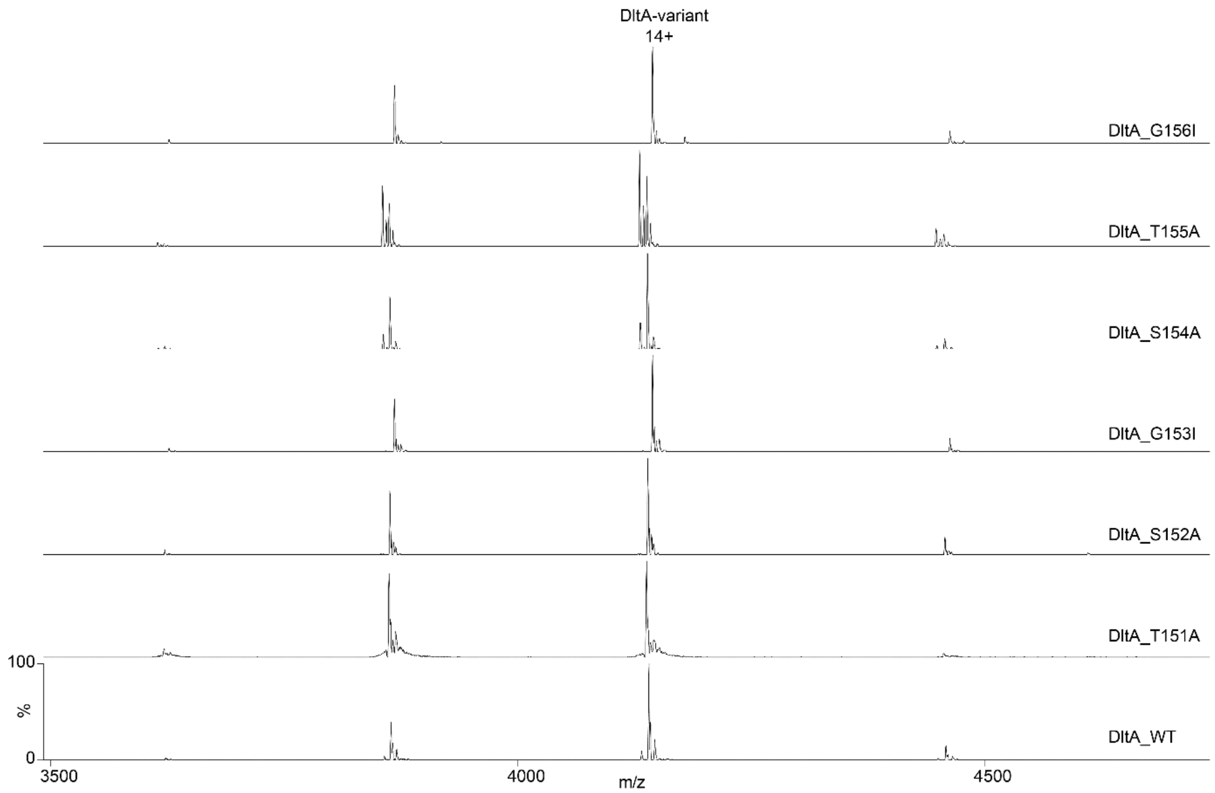

Figure S11: Native MS analyses of the purified DltA P-loop mutants. Mass spectra of DltA P-loop mutants. Heterogeneity in the case of S154A and T155A mutants is due to the presence of Tris. Theoretical and measured masses are listed in Table S2.

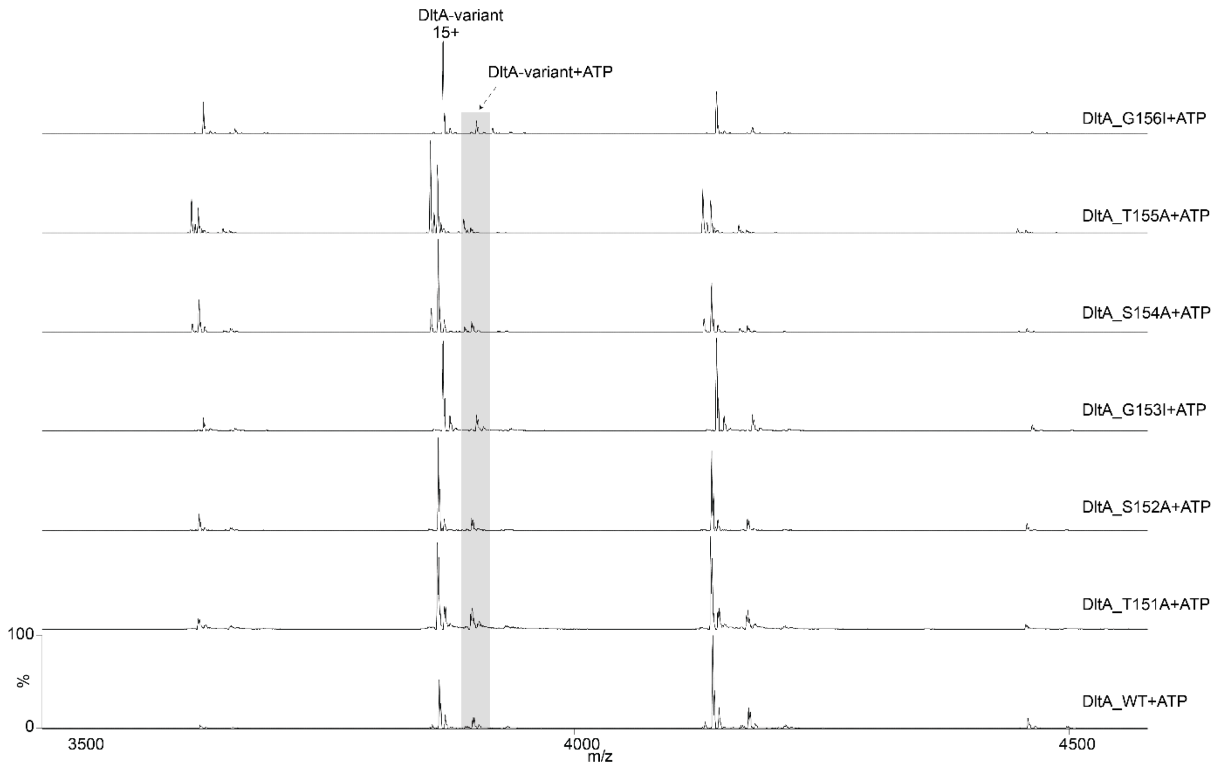

Figure S12: ATP binds non-covalently to all DltA P-loop mutants. Spectra of DltA mutants in the presence of ATP display their non-covalent binding abilities with ATP. Theoretical and measured masses are listed in Table S2.

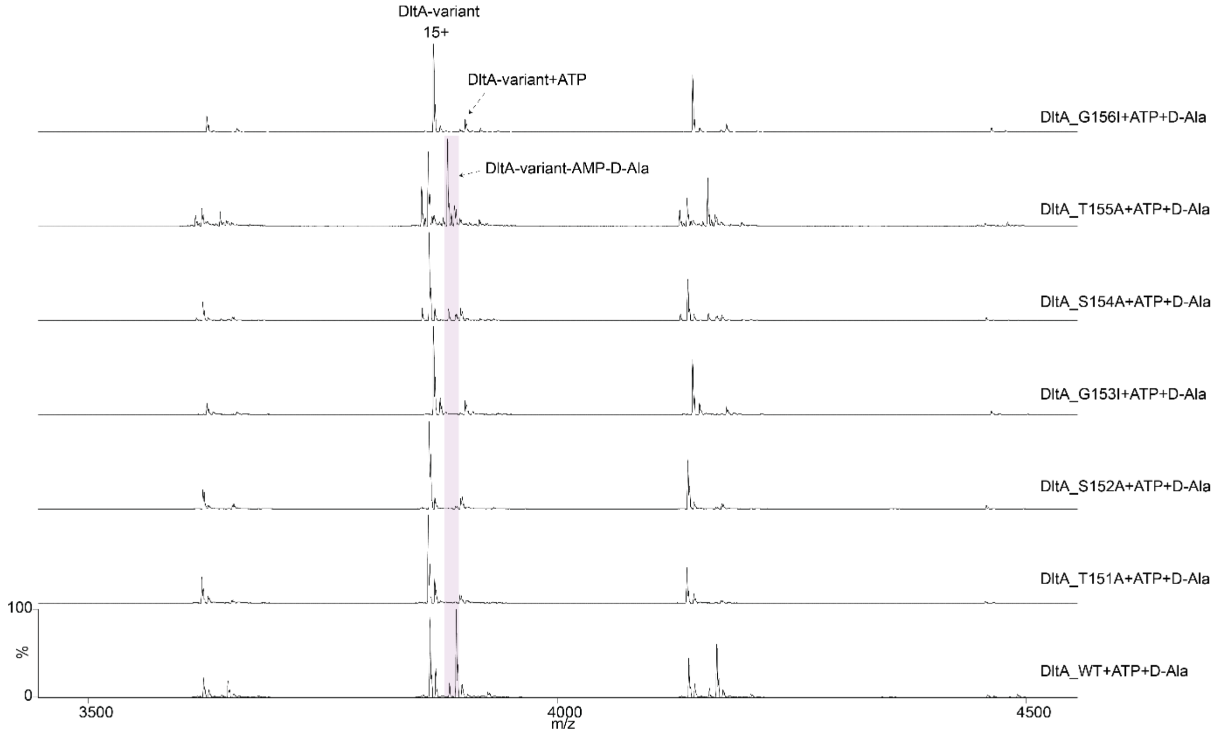

Figure S13: Adenylation activities of DltA mutants. Spectra of DltA mutants in the presence of ATP and D-Ala. AMP-D-Ala intermediate formation can only be observed clearly in the case of the wild type, S154A, and T155A, but with little to no in other cases. Theoretical and measured masses are listed in Table S2.

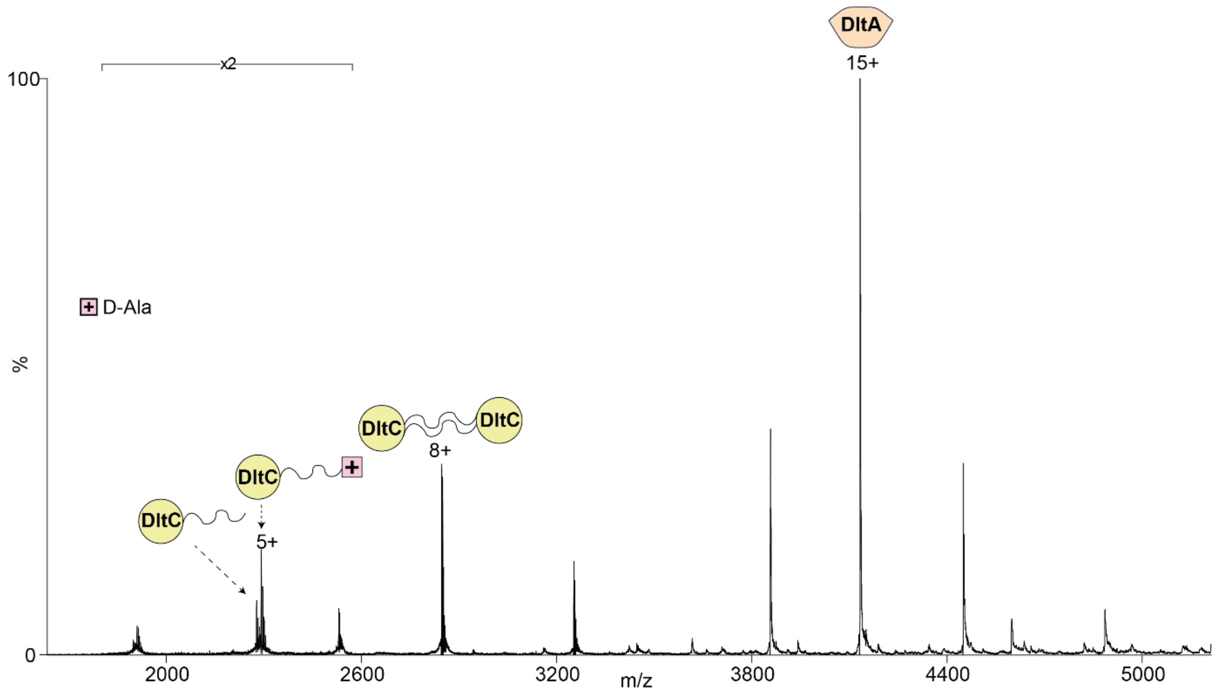

Figure S14: Transfer of D-Ala to DltC. Mass spectrum of the coupled reaction between DltA and DltC displays peaks of DltC with DltC-P-pant, DltC-P-pant-D-Ala, consistent with efficient transfer to D-Ala. Theoretical and measured masses are listed in Table S3.

Table S1: Sequences of primers used for generating DltA mutants.

**P-Loop mutants**	**T151A**	**Forward**	5’ atcatttatgcaagcggctcaacaggaaatccgaagggcgtc 3’
		**Reverse**	5’ tgttgagccgcttgcataaatgatatagaaggtttcgtgctcctt 3’
	**S152A**	**Forward**	5’ atttatacagccggctcaacaggaaatccgaagggcgtccag 3’
		**Reverse**	5’ tcctgttgagccggctgtataaatgatatagaaggtttcgtgctc 3’
	**G153I**	**Forward**	5’ tatacaagcatctcaacaggaaatccgaagggcgtccagatt 3’
		**Reverse**	5’ atttcctgttgagatgcttgtataaatgatatagaaggtttcgtg 3’
	**S154A**	**Forward**	5’ acaagcggcgcaacaggaaatccgaagggcgtccagatttca 3’
		**Reverse**	5’ cggatttcctgttgcgccgcttgtataaatgatatagaaggtttc 3’
	**T155A**	**Forward**	5’ agcggctcagcaggaaatccgaagggcgtccagatttcagcg 3’
		**Reverse**	5’ cttcggatttcctgctgagccgcttgtataaatgatatagaaggt 3’
	**G156I**	**Forward**	5’ ggctcaacaataaatccgaagggcgtccagatttcagcggcg 3’
		**Reverse**	5’ gcccttcggatttattgttgagccgcttgtataaatgatatagaa 3’
	**P158A**	**Forward**	5’ acaggaaatgcgaagggcgtccagatttcagcggcgaattta 3’
		**Reverse**	5’ ctggacgcccttcgcatttcctgttgagccgcttgtataaatgat 3’
	**K159A**	**Forward**	5’ ggaaatccggcgggcgtccagatttcagcggcgaatttacag 3’
		**Reverse**	5’ aatctggacgcccgccggatttcctgttgagccgcttgtataaat 3’

Table S2: Expected and measured masses for P-Loop mutants constructs

Construct	Calculated mass (Da)	Observed mass (Da)	Relevant figure
DltA WT	57842	57949±1.22	Fig S1, Fig S2
T151A	57811	57917±0.38	Fig 3, Fig 5, Fig S1, Fig S8, Fig S9
S152A	57825	57933±3.63	Fig 3, Fig 5, Fig S1, Fig S8, Fig S9
G153I	57897	58005±3.02	Fig 3, Fig 5, Fig S1, Fig S8, Fig S9
S154A	57825	57931±0.08	Fig 3, Fig 5, Fig S1, Fig S8, Fig S9
T155A	57811	57812±0.72<br>57920±0.27	Fig 3, Fig 5, Fig S1, Fig S8, Fig S9
G156I	57897	58006±1.54	Fig 3, Fig 5, Fig S1, Fig S8, Fig S9
DltA-T151A + ATP	58425	58447±0.3	Fig 3, Fig 5, Fig S8, Fig S10, Fig S11
DltA-S152A + ATP	58439	58440±0.33	Fig 3, Fig 5, Fig S8, Fig S10, Fig S11
DltA-G153I + ATP	58511	58511±0.03	Fig 3, Fig 5, Fig S8, Fig S10, Fig S11
DltA-S154A + ATP	57439	58440±0.35	Fig 3, Fig 5, Fig S8, Fig S10, Fig S11
DltA-T155A + ATP	58318	58318±0.13	Fig 3, Fig 5, Fig S8, Fig S10, Fig S11
DltA-G156I + ATP	58511	58512±0.91	Fig 3, Fig 5, Fig S8, Fig S10, Fig S11
DltA-S154A + AMP-D-Ala	58352	58441±0.69	Fig 3, Fig 5, Fig S8, Fig S10, Fig S11
DltA-T155A + AMP-D-Ala	58230	58230±0.65	Fig 3, Fig 5, Fig S8, Fig S10, Fig S11

Table S3: Expected and observed masses for protein reactions and products

**Construct**	**Calculated mass (Da)**	**Observed mass (Da)**	**Relevant figure**
DltA WT + ATP	58349	58456±0.17	Fig S4
DltA WT + D-Ala	57931	58137±5.87	Fig S4
DltA WT + AMP-D-Ala	58278	58367±0.09	Fig 2, Fig 3
DltA WT + Inhibitor	58259	58260±2.72	Fig 2, Fig S6
DltC Monomer	11042	11043±0.2	Fig 1, Fig S3
DltC + Ppant	11400	11380±0.32	Fig 1, Fig 4, Fig S3
DltC + Ppant-D-Ala	11471	11452±1.02	Fig 4, Fig 5

Unedited SDS-PAGE gel images that were used to make Supplementary Figure S1.

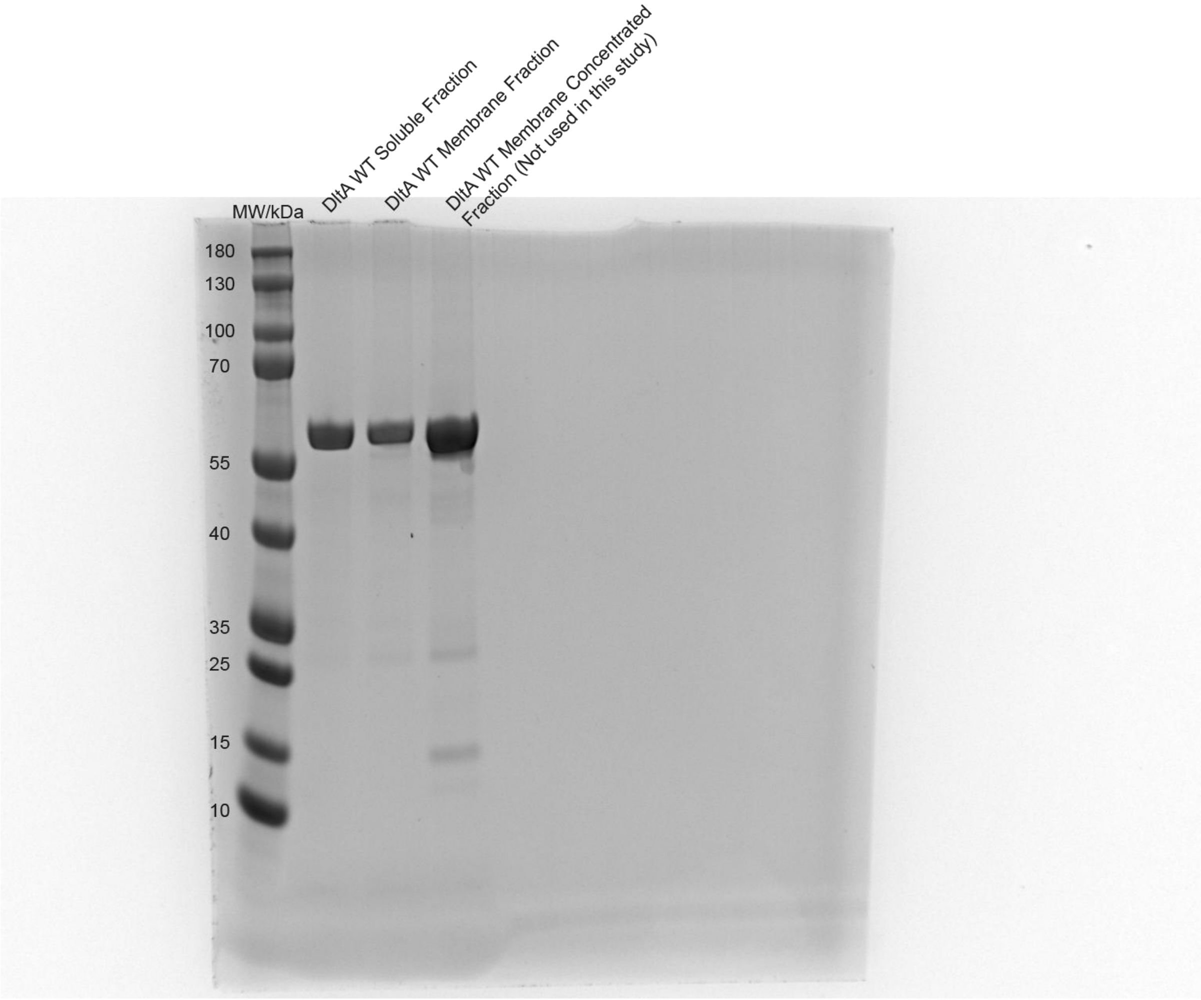

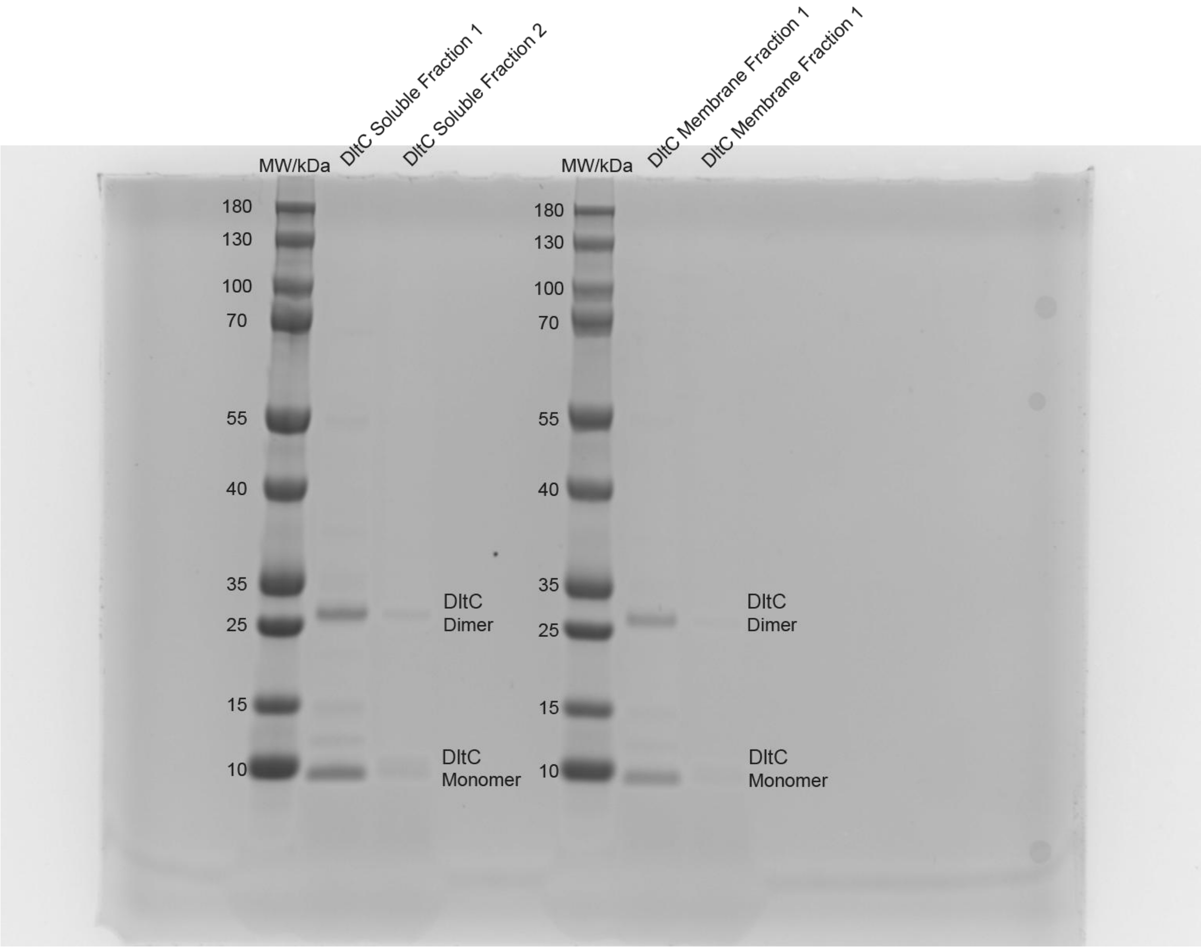
MW/kDa
DltC Soluble Fraction 1
DltC Soluble Fraction 2
MW/kDa
DltC Membrane Fraction 1
DltC Membrane Fraction 1
180
130
100
70
55
40
35
25
15
10
DltC Dimer
DltC Monomer
180
130
100
70
55
40
35
25
15
10
DltC Dimer
DltC Monomer

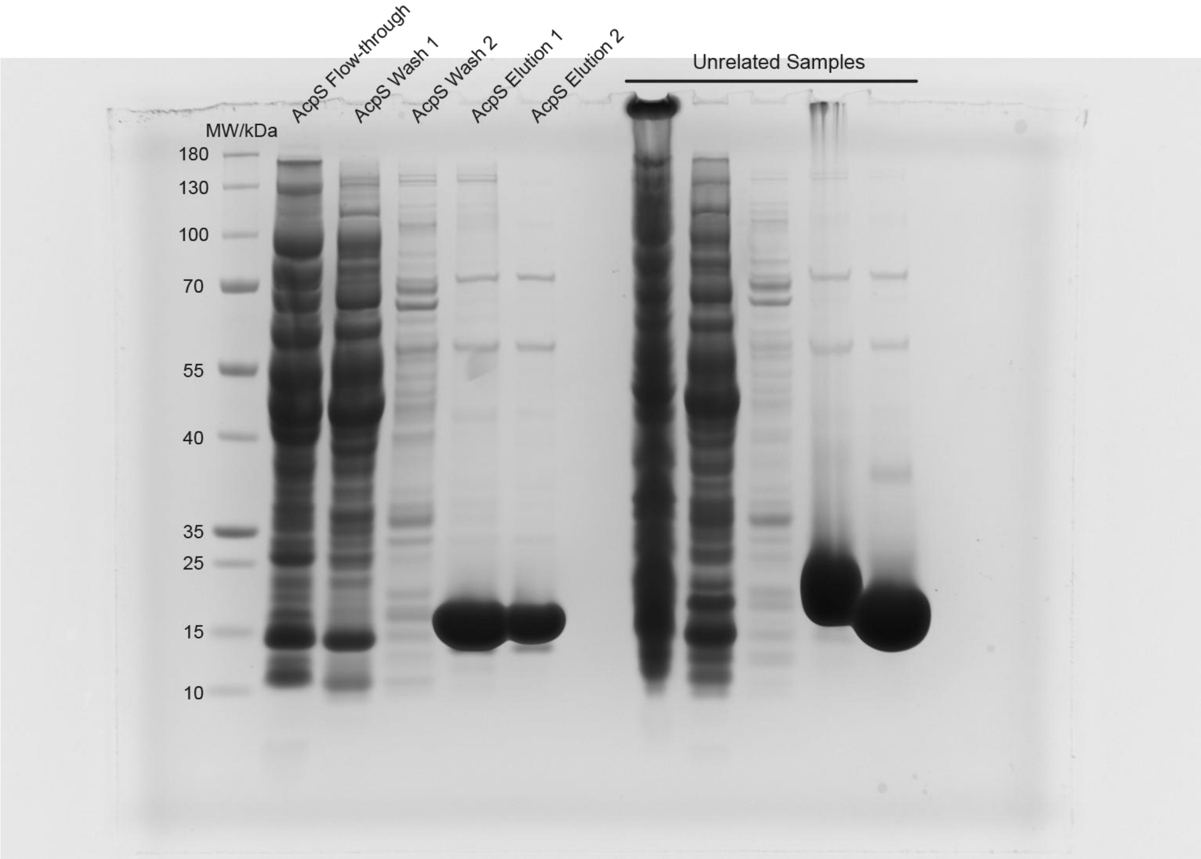
AcpS Flow-through
AcpS Wash 1
AcpS Wash 2
AcpS Elution 1
AcpS Elution 2
Unrelated Samples
MW/kDa
180
130
100
70
55
40
35
25
15
10

Unedited SDS-PAGE gel images that were used to make Supplementary Figure S10

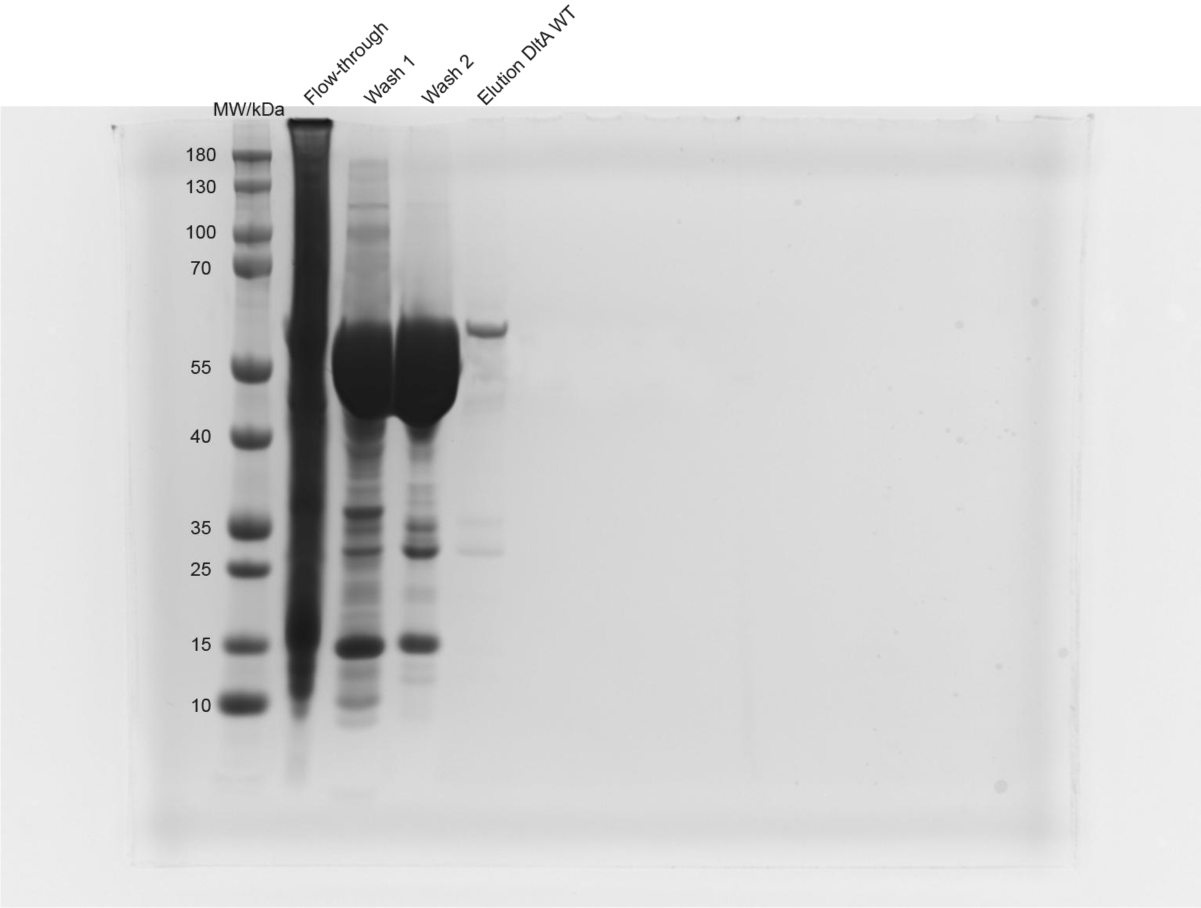

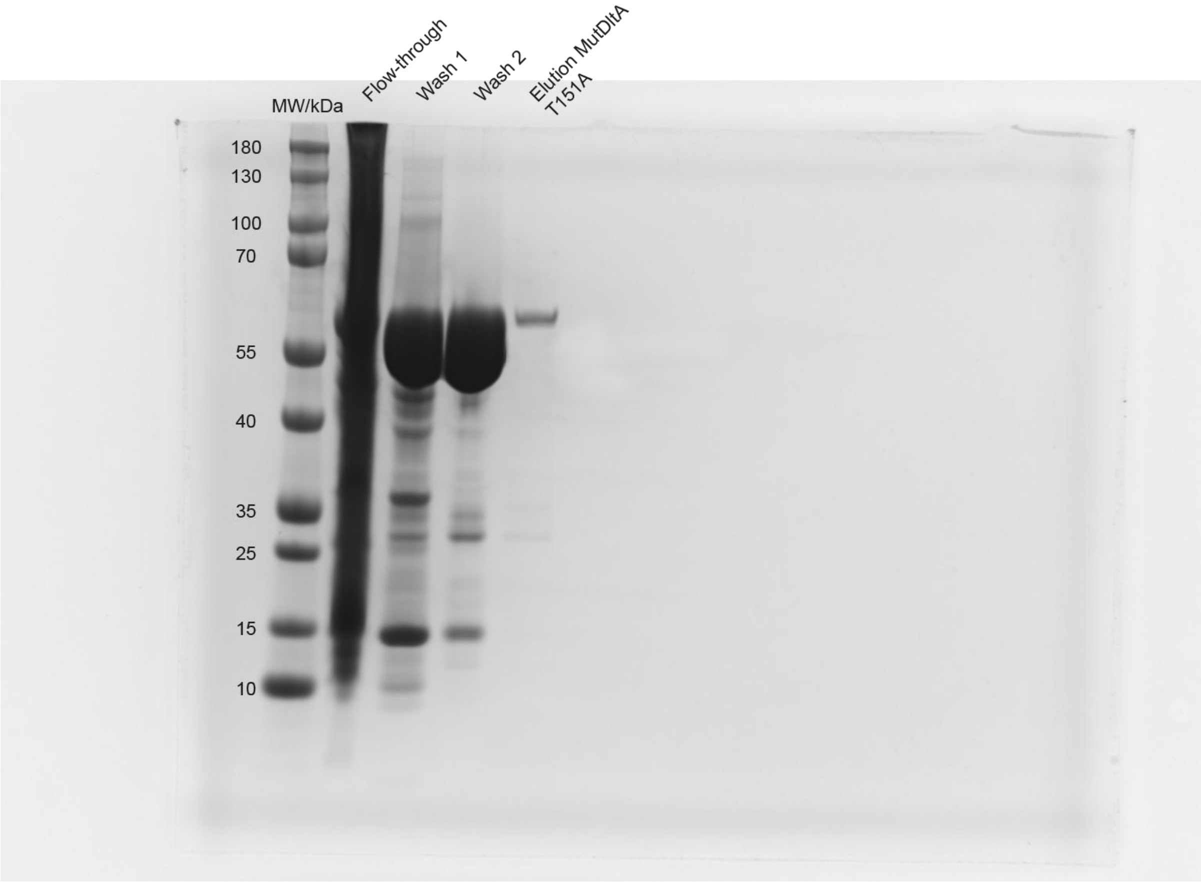
MW/kDa
Flow-through
Wash 1
Wash 2
Elution MutDltA T151A
180
130
100
70
55
40
35
25
15
10

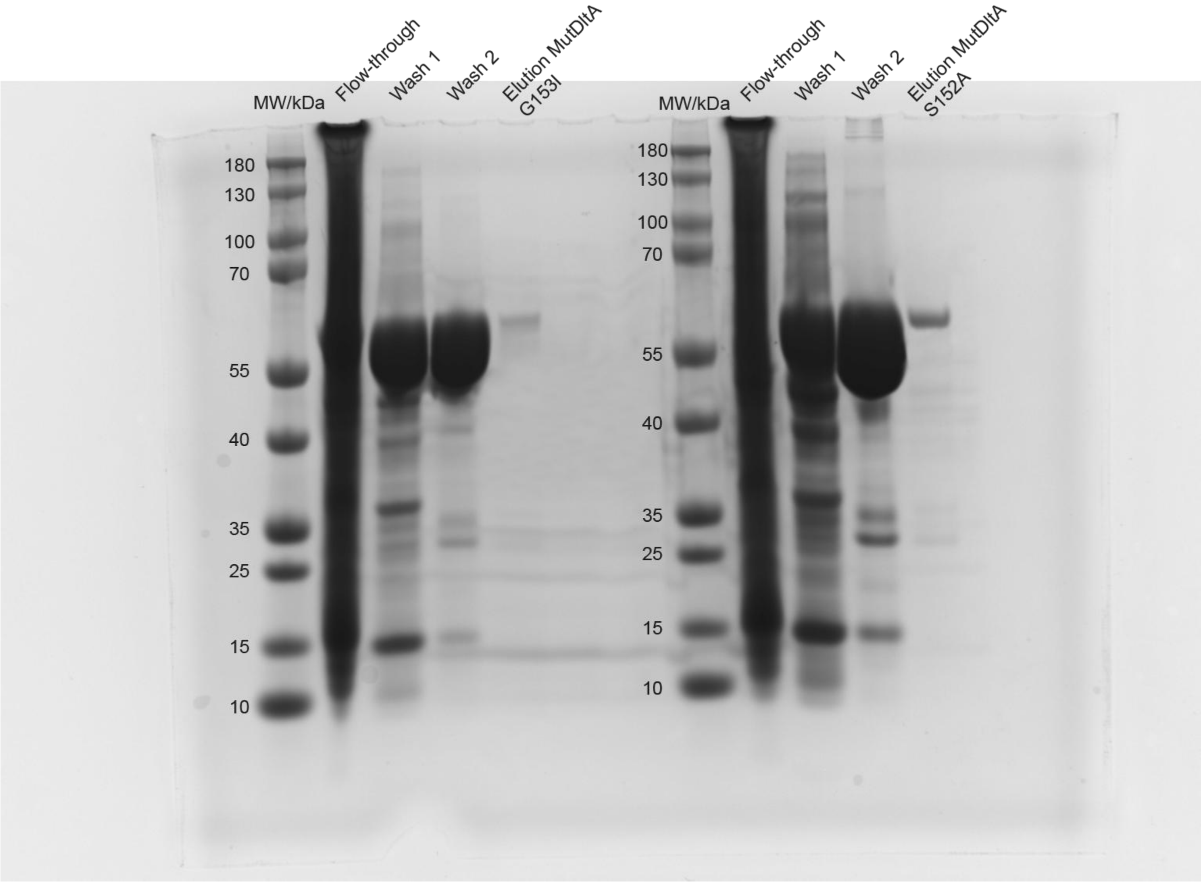
MW/kDa
Flow-through
Wash 1
Wash 2
Elution MutDltA G153I
180
130
100
70
55
40
35
25
15
10
MW/kDa
Flow-through
Wash 1
Wash 2
Elution MutDltA S152A
180
130
100
70
55
40
35
25
15
10

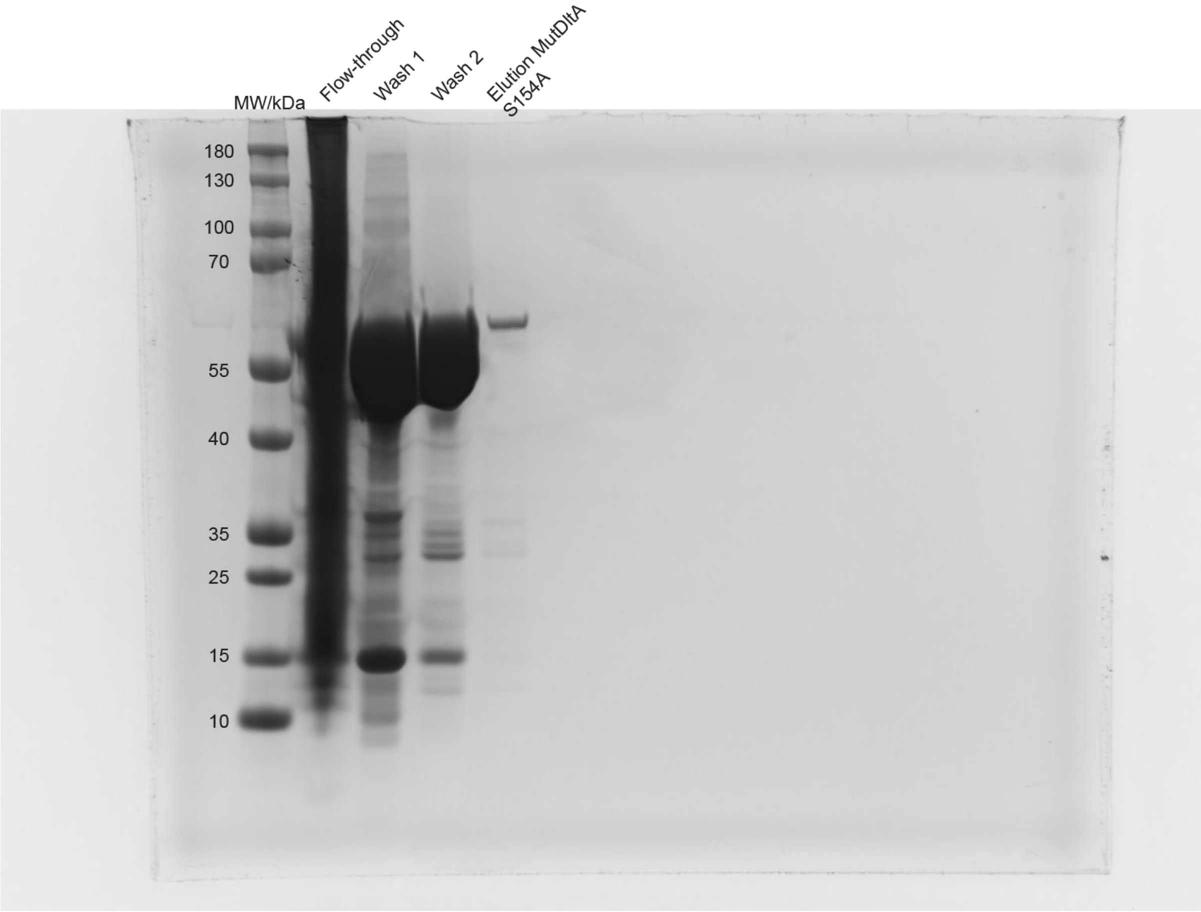
MW/kDa
Flow-through
Wash 1
Wash 2
Elution MutDltA S154A
180
130
100
70
55
40
35
25
15
10

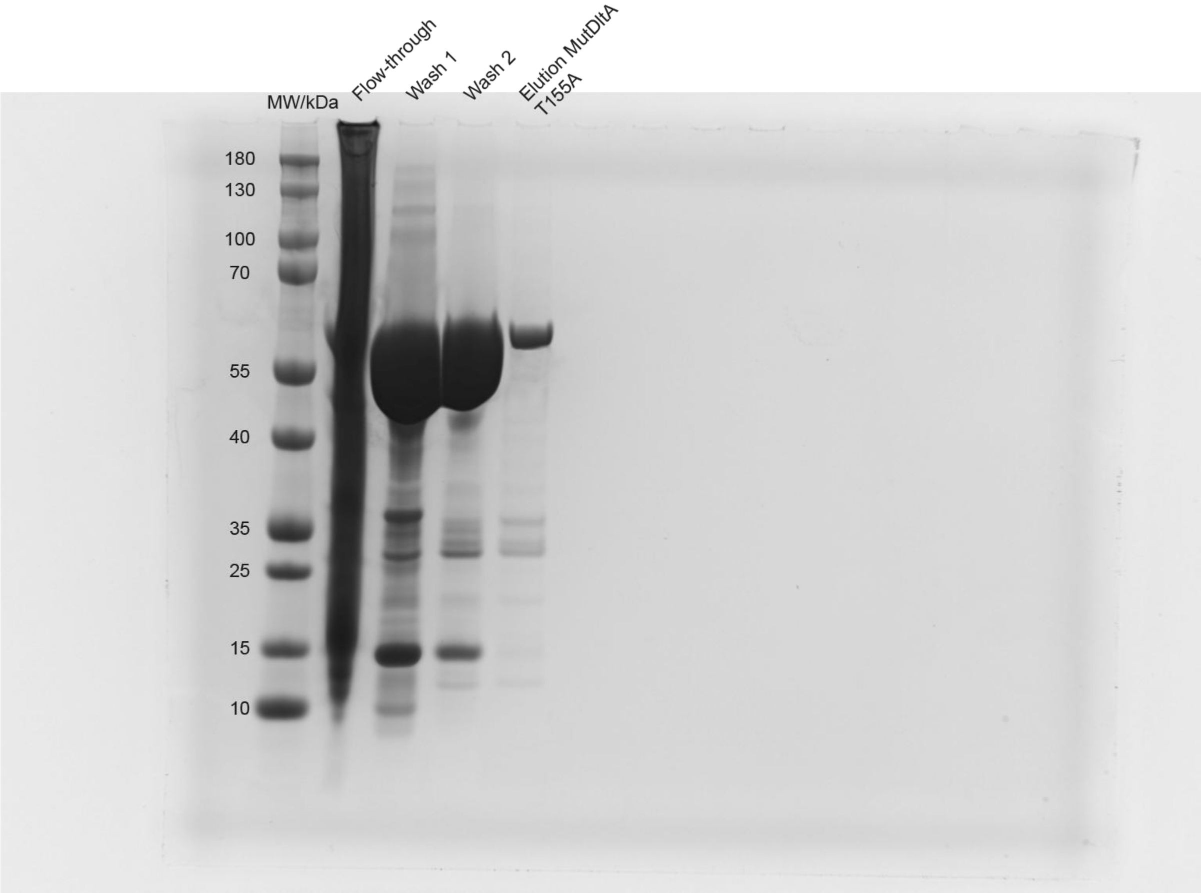
MW/kDa
Flow-through
Wash 1
Wash 2
Elution MutDltA T155A
180
130
100
70
55
40
35
25
15
10

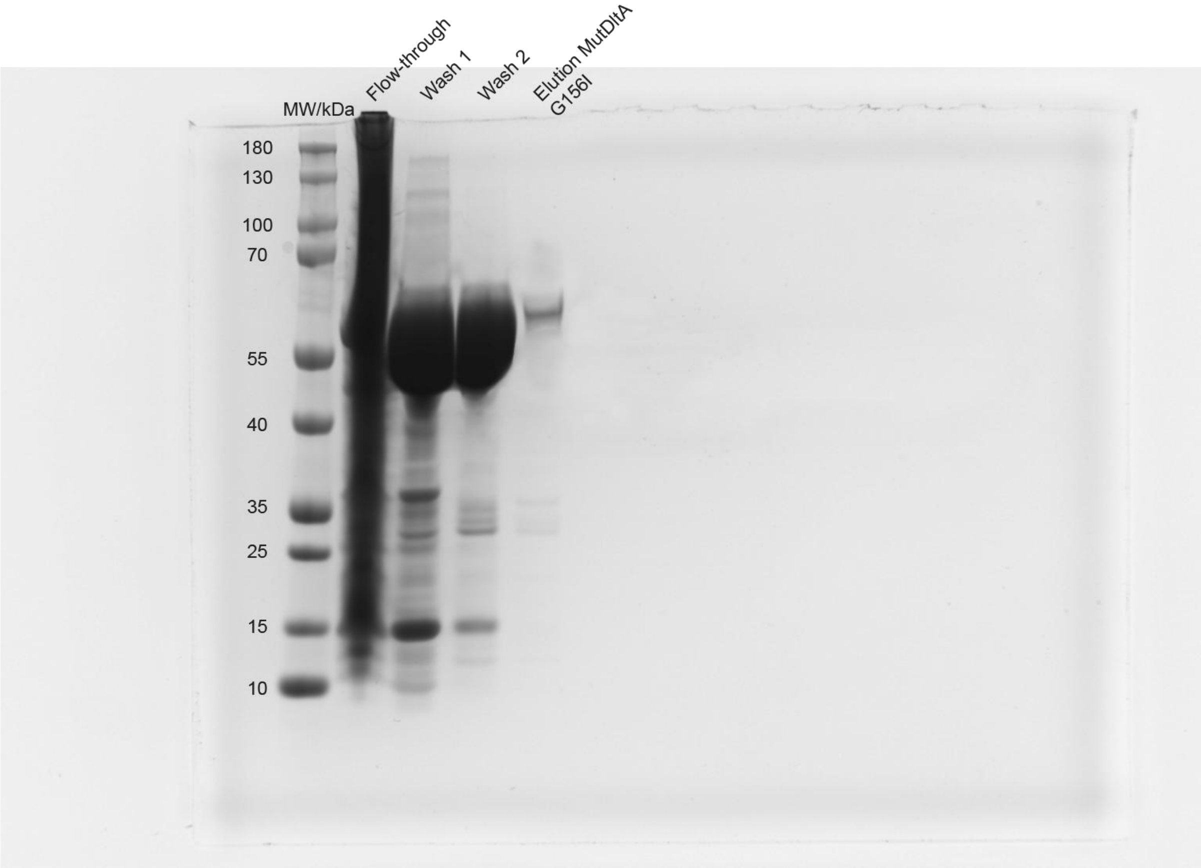
MW/kDa
Flow-through
Wash 1
Wash 2
Elution MutDltA G156I
180
130
100
70
55
40
35
25
15
10
